# Direct isolation of small extracellular vesicles from human blood using viscoelastic microfluidics

**DOI:** 10.1126/sciadv.adi5296

**Published:** 2023-10-06

**Authors:** Yingchao Meng, Yanan Zhang, Marcel Bühler, Shuchen Wang, Mohammad Asghari, Alessandra Stürchler, Bogdan Mateescu, Tobias Weiss, Stavros Stavrakis, Andrew J. deMello

**Affiliations:** ^1^ Institute for Chemical and Bioengineering, Department of Chemistry and Applied Biosciences, ETH Zürich, 8093 Zürich, Switzerland.; ^2^ Department of Neurology, University Hospital Zürich, 8091 Zürich, Switzerland.; ^3^ Clinical Neuroscience Center, University of Zürich, 8091 Zürich, Switzerland.; ^4^ Brain Research Institute, University of Zürich, 8057 Zürich, Switzerland.

## Abstract

Small extracellular vesicles (sEVs; <200 nm) that contain lipids, nucleic acids, and proteins are considered promising biomarkers for a wide variety of diseases. Conventional methods for sEV isolation from blood are incompatible with routine clinical workflows, significantly hampering the utilization of blood-derived sEVs in clinical settings. Here, we present a simple, viscoelastic-based microfluidic platform for label-free isolation of sEVs from human blood. The separation performance of the device is assessed by isolating fluorescent sEVs from whole blood, demonstrating purities and recovery rates of over 97 and 87%, respectively. Significantly, our viscoelastic-based microfluidic method also provides for a remarkable increase in sEV yield compared to gold-standard ultracentrifugation, with proteomic profiles of blood-derived sEVs purified by both methods showing similar protein compositions. To demonstrate the clinical utility of the approach, we isolate sEVs from blood samples of 20 patients with cancer and 20 healthy donors, demonstrating that elevated sEV concentrations can be observed in blood derived from patients with cancer.

## Teaser

Viscoelastic microfluidics enables label-free isolation of sEVs from human blood with high separation efficiency and recovery.

## INTRODUCTION

Extracellular vesicles (EVs) are present in all kinds of bodily fluids (including blood, saliva, and urine). They are a heterogeneous group of cell-derived lipid-based vesicles containing bioactive cargos, such as nucleic acids, proteins, and metabolites, and are normally classified into three main subtypes based on their size: small (sEVs; <200 nm), medium (mEVs; 200 to 800 nm) and large (lEVs; >800 nm) EVs (*1*–*3*). mEVs and lEVs are normally generated by the direct outward budding of the plasma membrane or cell apoptosis, while exosomes, which belong to the sEV subtype, are endosomal in origin (*4*, *5*). sEVs are present in all body fluids and thus serve as an excellent source of noninvasive diagnostic and prognostic biomarkers (*6*, *7*). Numerous studies have highlighted the importance of sEVs as biomarkers and therapeutic vectors for a number of diseases, including cancer, infections, and neurodegeneration (*8*–*10*). Conventional methods for sEV isolation, based on ultracentrifugation (UC), gradient centrifugation, and/or microfiltration centrifugation suffer from a range of limitations, including extended processing times, low yields (5 to 40%), low isolated sEV integrity, and high equipment costs (*11*–*14*). Specifically, UC, which is the gold-standard method for sEV isolation, often yields low exosome recoveries, with isolated sEVs being contaminated with coprecipitated protein aggregates. In addition, filtration methods exhibit are plagued by nonspecific protein binding to the filtration membrane and membrane blockage (*12*, *15*, *16*). Although a number of contemporary sEV isolation approaches, such as those based on precipitation (*17*–*19*), size exclusion chromatography (*20*, *21*), and immuno-affinity capture (*22*, *23*), have mitigated some of the aforementioned issues; they are far from ideal. For example, when using kit-based precipitation methods, coprecipitation of other nonexosomal contaminants, such as proteins and polymeric materials, is unavoidable (*12*, *24*). In addition, immunoaffinity and size exclusion chromatography–based capture methods almost always include elution steps, which result in sample loss, making both methods ill-suited for downstream analysis (*12*, *25*).

In the past decade, microfluidic technologies have been increasingly used as basic tools for sEV processing and isolation, due to their ability to precisely and controllably manipulate micrometer- and nanometer-sized objects (*26*, *27*). Depending on the mode of separation, microfluidic strategies for sEV isolation can be classified as being either passive or active in nature (*3*, *28*). Passive separation methods do not require the application of external forces but rather isolate sEVs through the use of size-dependent hydrodynamic forces (*29*–*33*) or complex channel structures [e.g., nanoporous membranes (*34*, *35*) and nanopillar arrays (*36*, *37*)]. Conversely, active separation methods require the application of external force fields, most notably acoustic (*38*–*40*), electric (*41*, *42*), and magnetic (*43*, *44*) fields to manipulate sEVs.

Active methods have been successfully used to separate blood-derived sEVs and are characterized by high isolation efficiencies. Through chip-based dielectrophoresis, Lewis *et al.* (*45*) successfully trapped sEVs (<500 nm in size) from whole blood and subsequently performed an on-chip immunofluorescence assay to detect the captured sEVs. While effective at isolating sEVs, the method is not able to isolate sEVs over an extended period of time, because the number of captured sEVs on the electrode array increases over time, causing a deterioration in separation efficiency. More recently, Sancho-Albero and co-workers successfully used magnetic Fe_3_O_4_ nanoparticles functionalized with antibodies to capture sEVs from whole blood (*43*). While the approach was able to isolate pancreatic cancer–derived exosomes from whole blood, immunocapture-based isolation involves antibody modification and conjugation steps, requires extra steps for releasing intact sEVs from the functionalized surface, and thus is difficult to perform in clinical environments. Using an entirely different strategy, Lee *et al.* (*46*) used standing ultrasound waves to exert differential acoustic forces on sEVs based on their size and density. Specifically, a pair of interdigitated transducer electrodes was used to generate standing surface acoustic waves across a sample flow, allowing isolation of exosomes from cell culture medium and erythrocyte-derived vesicles from blood, without labeling. Soon after, Huang and colleagues presented an elegant acoustofluidic device able to isolate extracellular vesicles directly from whole-blood samples (*38*). This device consists of a cell-removal module to remove large blood components, followed by an sEV-separation module to obtain sEVs with average diameters below 140 nm. Significantly, variation of fluid flow rates and the power of the RF signal applied to two pairs of interdigitated transducer electrodes allowed tunable isolation of sEVs based on size. Although this approach provides for outstanding sEV isolation efficiencies, both device fabrication and operation are complex, which again limits use in clinical settings.

Passive microfluidic systems incorporating physical filtration have also been successfully used to isolate sEVs from whole blood. For example Davies *et al.* (*47*) developed a microfluidic device integrating nanoporous membranes (based on porous polymer monoliths) for sEV and mEV (<500 nm) filtration from whole blood. Because these membranes can be easily blocked by other blood components, an electric field was used to realize electrophoretic separation in a cross-flow geometry. In addition, Chen and co-workers reported a multilayer membrane-integrated microfluidic device able to separate sEVs (<200 nm) from small volumes of whole blood (*48*, *49*). Here, a polycarbonate membrane is used to isolate sEVs isolation via stirring-enhanced filtration. Despite its obvious utility, the complex multilayer device architecture and need for pneumatically driven “micro stirrers” (to prevent device blockage) significantly limits its widespread applicability. Sunkara *et al.* (*50*) developed an alternative filtration platform, comprising two membrane filters with pore sizes of 20 and 600 nm, which is capable of extracting sEVs from small volumes of whole blood within 40 min. Unfortunately, the need to perform external centrifugation complicates its use in both research and clinical settings. Very recently, Li *et al.* (*51*) developed a cascaded microfluidic system, comprising a cell-removal circuit featuring with a 600-nm pore size membrane filter and an EV-isolation circuit incorporating a 20-nm pore size membrane filter, to direct isolate sEVs and mEVs from 10-fold diluted whole blood in 30 min. While the platform is successful in detecting protein biomarkers from blood-derived EVs, recovery rates are low (10.5%), and membrane fouling limits operational lifetime to a few min despite implementing pulsatile flow. In contrast to microfiltration methods, techniques based on passive hydrodynamic separation can also be used for blood-derived sEV isolation. For example, Tay *et al.* (*31*) reported an inertial microfluidic system, consisting of four multiplexed channels, that can isolate sEVs from whole blood in a passive and label-free manner. Although the reported device operated at high throughput (1.2 ml/hour of whole blood per channel), recovery rates were found to be unacceptably low (less than 20%). More recently, viscoelastic microfluidic systems, leveraging non-Newtonian fluids, have been shown to be simple yet effective tools for bioparticle manipulations and sEV isolation (*52*, *53*). For example, by adding small amounts of a biocompatible polymer, such as poly(ethylene oxide) (PEO), into both sample and guide flows, Liu *et al.* (*30*) successfully isolated sEVs and mEVs (with a size cutoff of 200 nm) from fetal bovine serum (FBS). Subsequently, the same group used a similar device to fractionate EVs from stage II breast cancer samples into three EV subgroups, namely, sEVs, mEVs, and lEVs (*54*). However, while exosomes could be isolated in a continuous, size-dependent, and label-free fashion, the system has yet to be used to process complex biofluids, such as blood. More recently, Asghari *et al.* (*29*) reported an oscillatory microfluidic device capable of differentially focusing sEVs and lEVs over relatively short channel lengths. Although efficient, the throughput of the system was relatively low due to the oscillatory nature of the flow and thus unsuitable for processing blood. In addition, Nam *et al.* (*32*) reported a sheathless viscoelastic microfluidic device able to separate sEVs (having an average diameter of 350 nm) from whole blood. Although large micrometer-scale blood components, including red blood cells (RBCs), white blood cells (WBCs), and platelets (PLTs), could be efficiently removed, smaller biospecies (with average diameters less than 1 μm) were distributed amongst all outlets, yielding low separation efficiencies and recoveries.

**Fig. 1.**
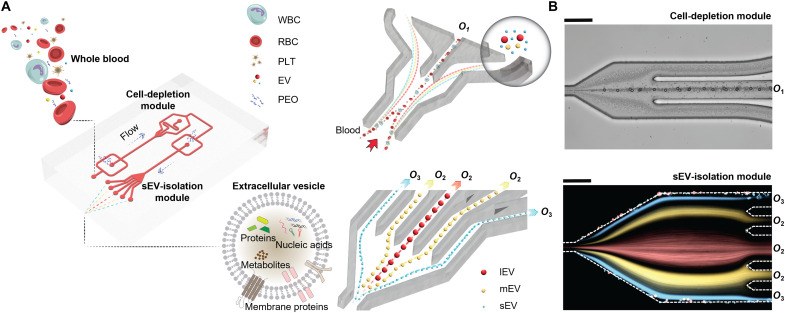
Schematic illustration of the viscoelastic microfluidic system for sEV separation from whole blood. (**A**) The microfluidic device consists of two sequential modules: a cell-depletion module and an sEV-isolation module. Blood components larger than 1 μm, including WBCs, RBCs, and PLTs, are first removed from outlet *O*_1_ in the cell-depletion module, and, subsequently, the cell-free blood sample flows downstream into the sEV-isolation module, where lEVs and mEVs are collected from outlet *O*_2_, while sEVs are collected from outlet *O*_3_. (**B**) Images of size-based particle separations in each isolation module. The bright-field image shows the separation of 1 μm from 3-, 7-, and 12-μm particles in the cell-depletion module. The 3-, 7-, and 12-μm particles are focused along the centerline and exit through the waste outlet *O*_1_, whereas 1-μm particles are located near the sidewalls, flowing downstream into the sEV-isolation module. The fluorescence image is a composite image of 1-μm (red)–, 500-nm (yellow)–, and 100-nm (blue)–sized particle trajectories in the sEV-isolation module. The 1-μm and 500-nm particles are collected at outlet *O*_2_, while the 100-nm particles are collected at the outlet *O*_3_. Scale bars, 100 μm.

Within clinical settings, there is an urgent need for EV isolation systems that are inexpensive to manufacture, simple to operate, require no specialized expertise, involve minimal sample preparation, and yield consistent and reproducible results, all while delivering high separation purities and recovery rates. The aforementioned “active” techniques for separating blood-derived sEVs require the generation and application of external forces, which necessitates the use of external equipment (*43*, *45*, *46*). This, in turn, increases processing costs and complicates experimental workflows. Expectedly, passive microfluidic methods for isolating sEVs from whole blood have been shown to be advantageous with regard to cost, ease of use, and reproducibility, but they are not without limitations. For example, filtration-based platforms are susceptible to membrane clogging (*47*–*51*), with other passive techniques, such as those based on inertial and viscoelastic microfluidics (*31*, *32*), exhibiting suboptimal recoveries and purities. In clinical contexts, alongside considerations relating to affordability and user-friendliness, the importance of achieving high levels of sEV recovery and purity is of paramount significance, especially in situations where minimal quantities of biofluid are available. Accordingly, there is an unmet need for efficient, simple, and low-cost microfluidic tools that are able to isolate sEVs from whole blood in a rapid, reproducible, and efficient manner. To address this gap, we here present a passive high-efficiency, viscoelastic, and label-free microfluidic device capable of isolating sEVs directly from human blood. The integrated system comprises two modules: a cell-depletion module and a sEV-isolation module (Fig. 1A). In the first module, micrometer-scale blood components, notably WBCs, RBCs, and PLTs, are efficiently removed from the blood sample. Subsequently, the now “cell-free” blood is seamlessly introduced into the second module, where sEVs are separated and isolated from other EV subgroups. To demonstrate the efficacy of the system in clinical liquid biopsies, we isolate sEVs from the blood of 20 patients with cancer (CPs) and 20 healthy donors (HDs) and compare their concentrations and sizes with data obtained via gold-standard UC. Significantly, we also use our device to monitor the concentration and size of sEVs from 10 HDs as a function of storage time. Compared with current sEV isolation techniques, the presented viscoelastic microfluidic platform provides a low-cost and easy-to-operate approach with high levels of sEV recovery and purity, engendering sEV research in both research and clinical settings.

## RESULTS

### Chip design and the working principle for viscoelastic sEV separation

Figure 1 presents the structure of the microfluidic device and the underlying mechanism of sEV separation from whole blood. Briefly, the device consists of two modules arranged in sequence: a cell-depletion module and an sEV-isolation module. All fluidic structures are of a constant height of 55 μm (fig. S1). As shown in Fig. 1A, whole blood mixed with a PEO solution is introduced via inlet *I*_1_, while inlets *I*_2_ and *I*_3_ are used to inject guide fluid. In the first section, blood components larger than 1 μm, including PLTs, RBCs, and WBCs, are eliminated and collected from outlet *O*_1_. Subsequently, the now cell-free fluid (i.e., plasma) flows downstream into the sEV-isolation module, where larger EVs, including mEVs and lEVs, are collected from *O*_2_, while sEVs are collected from *O*_3_. Figure S2 schematically depicts the mechanism by which small species are separated from larger species, based on size-dependent forces. Micrometer- and nanometer-sized species within a viscoelastic medium primarily experience three size-dependent forces: elastic forces (***F***_e_ ∝ *a*^3^), viscous drag (***F***_d_ ∝ *a*), and inertial lift forces (***F***_i_ ∝ *a*^4^), where *a* is the particle diameter. When entering the cell depletion module, species of all sizes are initially aligned close to the channel sidewalls. As they move along the channel, size-dependent forces act on the contained species, leading to distinct lateral migration paths. By controlling the volumetric flow rates of both sample and guide flows, larger species can be made to migrate rapidly to the centerline, while smaller species exhibit little lateral displacement and remain close to the sidewalls. In this manner, and under optimized sample and guide flow rates, species may be separated on the basis of size. Figure 1B presents particle trajectories observed in each isolation module. In the bright-field image, 3-, 7-, and 12-μm particles are focused along the centerline and exit through the waste outlet *O*_1_, whereas 1-μm particles remain near the sidewalls and flow downstream into the sEV-isolation module. The composite fluorescence image reports particle trajectories for 1-μm (red), 500-nm (yellow), and 100-nm (blue) particles within the sEV-isolation module. In this module, 1-μm and 500-nm particles are collected at outlet *O*_2_, while the 100-nm particles are collected via outlet *O*_3_.

### Theoretical analysis

**Fig. 2.**
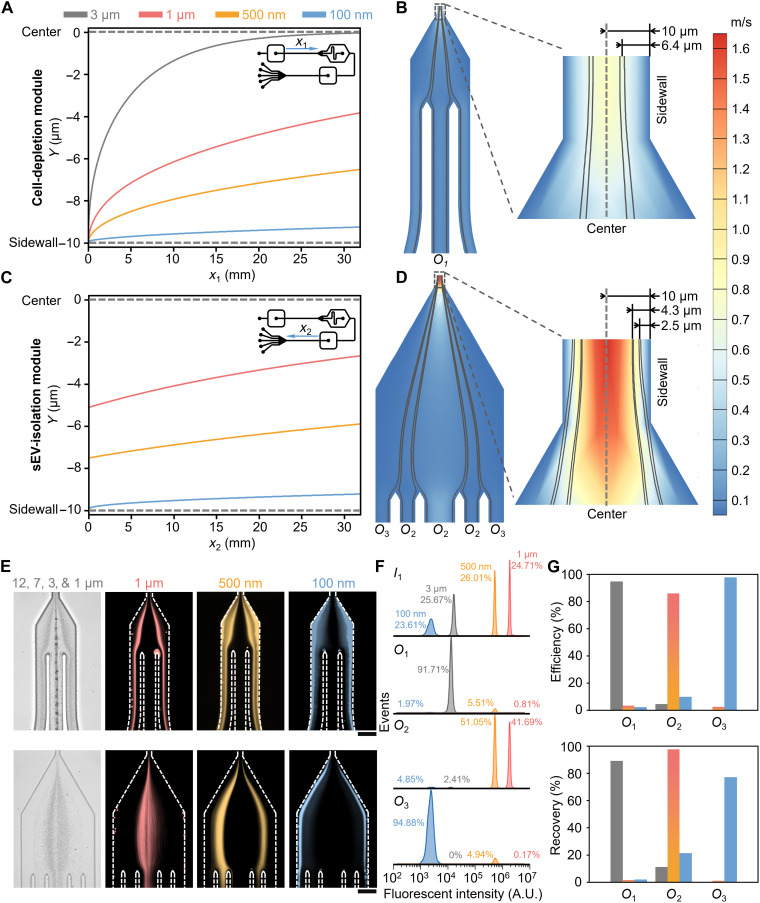
Theoretical prediction and experimental analysis of particle separation in the PEO solution. (**A**) Calculated particle trajectories for 3-μm, 1-μm, 500-nm, and 100-nm particles in the cell-depletion module. The 3-μm particles gradually migrate from the sidewalls to the centerline, while the rest migrate to different lateral positions. (**B**) Simulated flow field distribution at the separation section of the cell-depletion module. Streamline analysis indicates that at a distance of more than 6.4 μm from the sidewall, 3-μm particles are directed to *O*_1_. (**C**) Calculated particle trajectories in the sEV-isolation module. The 1-μm, 500-nm, and 100-nm particles enter the sEV-isolation module at different lateral positions and gradually migrate to different focusing positions. (**D**) Simulated flow field distribution at the separation region of the sEV-isolation module. Streamline analysis shows that, at distances less than 2.5 μm from the sidewalls, 100-nm particles will exit through *O*_3_. (**E**) Particle trajectories in the separation sections of both cell-depletion (top row) and sEV-isolation (bottom row) modules. In the cell-depletion module (top row), 12-, 7-, and 3-μm particles are focused along the centerline and exit through *O*_1_, whereas smaller particles are located near the sidewalls. In the sEV-isolation module (bottom row), 1-μm and 500-nm particles are collected at *O*_2_, while 100-nm particles are collected at *O*_3_. (**F**) Flow cytometry analysis of particle size distributions for the mixture at inlet *I*_1_ and fluid collected at each outlet (*O*_1_, *O*_2_, and *O*_3_). At *O*_3_, the purity of the 100-nm-diameter particles reaches 95%. A.U., arbitrary units. (**G**) Separation efficiencies and recoveries calculated from flow cytometry data. At *O*_3_, after separation, 100-nm-diameter particles make up over 97% of all collected particles, with recoveries reaching 77%. Scale bars, 100 μm.

A numerical model was developed in COMSOL to predict particle trajectories within a viscoelastic medium flowing through the microfluidic device. A detailed description of the model is provided in Materials and Methods. The model was used to investigate the trajectories of particles of various sizes: 3-μm, 1-μm, 500-nm, and 100-nm diameters, which are representative of PLTs, lEVs, mEVs, and sEVs, respectively. At low PEO concentrations, the viscosity of the carrier fluid is independent of shear rate between 10 and 1000 s^−1^ (fig. S3) and thus can be described using the Oldroyd-B model within the simulation (*30*). Figure 2A presents particle trajectories within the cell-depletion zone (along *x*_1_), with Figure 2C reporting particle trajectories in the sEV-isolation zone (along *x*_2_). In addition, Fig. 2 (B and D) presents flow field distributions in the expansion regions close to the outlet channels. Sample entering through *I*_1_ and both guide fluids entering through *I*_2_ and *I*_3_ contained 0.1, 0.15, and 0.1% w/v 600-kDa PEO concentrations, respectively. These concentrations were chosen on the basis of previous studies showing that high-resolution separation of micrometer-sized species is realized when sample and guide fluids have different viscosities (*55*, *56*). The flow rates for inlets *I*_1_, *I*_2_, and *I*_3_ were set to 200, 2000, and 3000 μl/hour, respectively. As shown in Fig. 2A, the 3-μm-diameter particles gradually migrate from the sidewalls to the centerline, while the 1-μm, 500-nm, and 100-nm particles migrate to different lateral positions between the sidewall and centerline. Streamline analysis shows that particles migrating more than 6.4 μm from the sidewall, the 3-μm-diameter particles in the current case, are directed to outlet *O*_1_, with the 1-μm, 500-nm, and 100-nm particles exiting the cell depletion module via the side outlet *O*_3_ (Fig. 2B). As shown in Fig. 2C, the 1-μm, 500-nm, and 100-nm particles enter the sEV-isolation module (*x*_2_ = 0) at different lateral positions, approximately 0.1, 2.5, and 4.9 μm from the sidewall for 100-nm, 500-nm, and 1-μm particles, respectively (fig. S4), and gradually migrate from the sidewalls to different positions in the channel that lead to outlets *O*_2_ and *O*_3_. In the sEV-isolation module, as shown in Fig. 2D, for a distance less than 2.5 μm from the sidewalls, 100-nm particles (representatives of sEVs), exit through the side outlet (marked “*O*_3_”), while the 1-μm and 500-nm-diameter particles exit through the central outlet (marked “*O*_2_”). In summary, the simulation results indicate that species of various sizes (3 μm, 1 μm, 500 nm, and 100 nm) exit from distinct outlets using the proposed method under defined conditions. These findings are supported by the experimental data presented in the subsequent sections. A detailed discussion regarding the influence of other geometrical parameters (fig. S5), guide flow rates (fig. S6), and sample flow rates (fig. S7) on particle separation performance is provided in text S1.

### Isolation of 100-nm-diameter particles from a complex mixture

The separation performance of the device was initially examined using polystyrene (PS) particles introduced at inlet *I*_1_. PS particles had average diameters of 12 μm, 7 μm, 3 μm, 1 μm, 500 nm, and 100 nm, which closely correspond to the characteristic dimension of WBCs, RBCs, PLTs, lEVs, mEVs, and sEVs, respectively. Both the sample and guide fluids were suspended in 1× phosphate-buffered saline (PBS) containing 600-kDa PEO. PEO concentrations for the fluids at inlets *I*_1_, *I*_2_, and *I*_3_ were 0.1, 0.15, and 0.1% w/v, respectively. To determine the optimal flow parameters, we measured the particle trajectories of 500-nm diameter particles, because they represent the cutoff between smaller particles (100 nm) and larger particles (1, 3, 7, and 12 μm). Figure S8 presents trajectory data for various guide and sample flow rates, with the aim of collecting particles through the outlets marked by yellow arrows. It can be seen that, when the flow rates at *I*_1_, *I*_2_, and *I*_3_ were 200, 2000, and 3000 μl/hour, respectively, 500-nm-diameter particles exit through the desired outlets. In addition, Fig. 2E shows the size-dependent separation of 12-μm, 7-μm, 3-μm, 1-μm, 500-nm, and 100-nm PS particles in the two-module device under the optimized flow conditions. As shown in Fig. 2E, 12-, 7-, and 3-μm particles were imaged in bright-field mode, with the particle trajectories of 1-μm, 500-nm, and 100-nm diameter being observed using fluorescence imaging. Within the cell-depletion module (Fig. 2E, top row), the large particles (12, 7, and 3 μm) are focused along the centerline and exit through outlet *O*_1_, whereas small particles (1 μm, 500 nm, and 100 nm) move along streamlines close to sidewalls and are thus able to enter the sEV-isolation module. Within the sEV-isolation module (Fig. 2E, bottom row), the 1-μm- and 500-nm-diameter particles are directed toward the outlet *O*_2_, while the 100-nm-diameter particles are collected via outlet *O*_3_. To better quantify separation performance, the compositions of the sample (i.e., 3-μm, 1-μm, 500-nm, and 100-nm PS particles) delivered to the inlet and the samples collected from each outlet (*O*_1_, *O*_2_, and *O*_3_) were analyzed by flow cytometry. For simplicity, 12-μm and 7-μm particles were not included in the flow cytometry analysis because the bright-field data in Fig. 2E confirm their effective removal. Figure 2F reports fluorescence intensity distributions for the particle mixture entering inlet *I*_1_ and for aliquots collected at each outlet (*O*_1_, *O*_2_, and *O*_3_). It can be seen that the sample entering the microfluidic devices contains approximately equal proportions of the 3-μm (25.67%)–, 1-μm (24.71%)–, 500-nm (26.01%)–, and 100-nm (23.61%)–diameter particles. Conversely, fluid exiting through outlet *O*_1_ primarily contains 3-μm-diameter particles (91.71%), with a minor percentage of 1-μm particles (0.81%), 500-nm particles (5.51%), and 100-nm particles (1.97%). This analysis confirms that the cell-depletion module efficiently removes the vast majority of species with diameters above 3 μm. Analysis of fluid exiting through outlet *O*_2_ indicates a similar percentage of 500-nm- and 1-μm-diameter particles (51.05 and 41.69%, respectively), with much smaller proportions of 3-μm- and 100-nm-diameter particles (4.85 and 2.41%, respectively). Last, analysis of fluid exiting through outlet *O*_3_ demonstrates that 94.88% of particles have a diameter of 100 nm, with the remaining 5.11% comprising 1-μm and 500-nm-diameter particles. These data confirm the passive and highly efficient separation of 100-nm particles from a complex mixture under continuous flow. Figure 2G presents separation efficiency and recovery data for 3-μm, 1-μm, 500-nm, and 100-nm particles collected at all outlets. As shown in Fig. 2G (top), approximately 95% of particles collected from outlet *O*_1_ are 3 μm in diameter, 85% of particles with diameters at 1 μm and 500 nm are collected from outlet *O*_2_, and more than 97% of the particles collected at the side outlet *O*_3_ are 100 nm in diameter. In addition, and as shown in Fig. 2G (bottom), nearly 90% of particles recovered from the outlet *O*_1_ have diameters larger than 3 μm. Last, a recovery of 97% was achieved for 1-μm and 500-nm particles at outlet *O*_2_, and a recovery of 77% is observed for 100-nm particles (representatives of sEVs) at outlet *O*_3_. Definitions of “purity,” “recovery,” and “separation efficiency” are provided in text S2.

To study the influence of PEO molecular weight on the separation efficiency, we additionally measured particle trajectories for a solution 1-MDa (rather than 600-kDa) PEO (fig. S9). Under these conditions, particles with diameters of 500 and 100 nm are now distributed across the whole width of the sEV-isolation module, leading to inefficient particle separation. We also attempted to separate the particle mixture without the addition of any PEO (fig. S10). Under these conditions, the 1-μm, 500-nm, and 100-nm particles cannot be separated under all flow rate conditions.

### Direct isolation of sEVs from human blood

To more completely assess the performance of the microfluidic device in real-world applications, we next studied the isolation of sEVs from human blood. We initially examined the effect of the blood dilution ratio, γ, the volumetric ratio of 1× PBS (supplemented with 0.1% w/v 600-kDa PEO) to whole blood, on separation performance (fig. S11). For all dilution ratios in fig. S11A, blood cells are focused toward the channel centerline (rather than remaining near the channel walls). However, as the dilution ratio is decreased, a larger number of blood cells are present at the side channels, and the focusing width increases. This means that more cells will be transferred from the cell-depletion module to the sEV-isolation module. Figure S11B evaluates the RBC removal rate from outlet *O*_1_. As the blood dilution ratio increases, a higher number of RBCs are removed from outlet *O*_1_, indicating an improved separation efficiency for RBCs. At both 4:1 and 8:1 dilution ratios, a comparable RBC removal rate is observed, with over 99% of RBCs being effectively eliminated. With a view to balancing separation efficiency with whole blood throughput, a blood dilution ratio of 4:1 was considered optimal and adopted in subsequent experiments. Using these conditions, we next spiked a mixture of PS particles (with diameters of 3 μm, 1 μm, 500 nm, and 100 nm) into whole blood. As shown in fig. S12, the separation efficiency and recovery values for the 100-nm particles (mimicking sEVs) in outlet *O*_3_ were in excess of 96 and 72%, respectively.

**Fig. 3.**
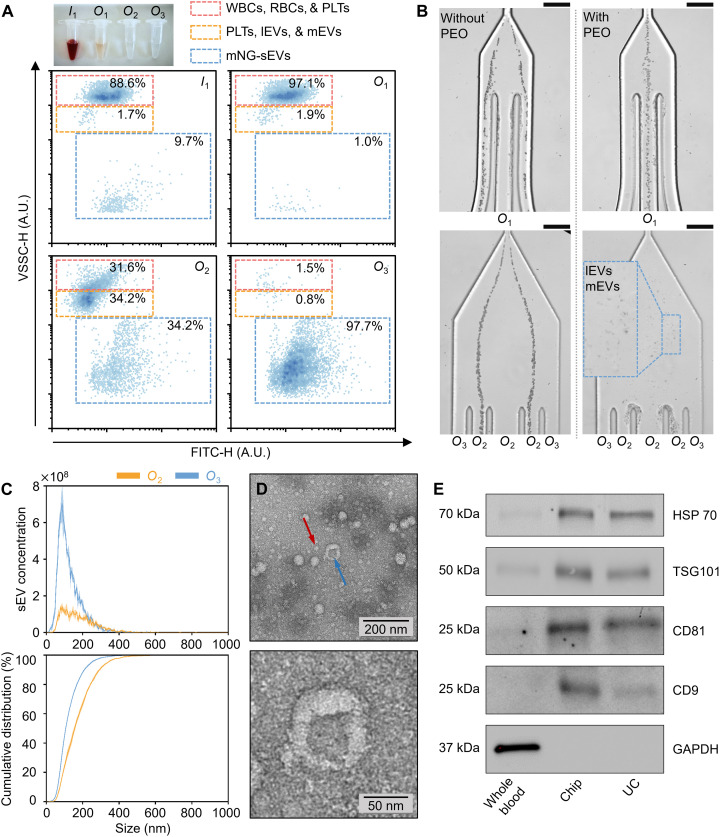
Characterization of sEV isolated from whole blood using the microfluidic device. (**A**) Particle separation results derived from scatter plots of VSSC-H versus FITC-H. Initially, 9.7% of mNG-sEVs (approximately 5 × 10^8^ species/ml of whole blood) were spiked in blood. Almost all WBCs and RBCs (97%), and a vast majority of PLTs were efficiently removed in the cell-depletion module from *O*_1_, while lEVs, mEVs, and remaining blood cells are further depleted in the sEV-isolation module. This leads to the extraction of spiked mNG-sEVs from *O*_3_ with a purity above 97%. (**B**) Comparison of particle trajectories in blood in the absence (left column) and presence (right column) of PEO. When blood is supplemented with PEO (right), the vast majority of WBCs, RBCs, and PLTs can be removed from the cell-depletion module, and the remaining components enter the sEV-isolation module where lEVs and mEVs are collected at *O*_2_. (**C**). NTA was performed for samples collected from *O*_2_ (*n* = 3 independent technical replicates, means ± SD) and *O*_3_ (*n* = 3 independent technical replicates, means ± SD) to determine the size distribution and the concentration of sEVs (top) and their cumulative distribution (bottom). sEV concentration is much higher for fluid collected from *O*_3_ than for fluid collected from *O*_2_, with the cumulative distribution indicating efficient removal of larger EVs from *O*_2_. (**D**) Transmission electron microscopy (TEM) images of isolated sEVs. The blue arrow marks a typical sEV particle, and the red arrow marks lipoprotein. (**E**) Western blotting (WB) results for EV (HSP70, TSG101, CD81, and CD9) and non-EV [glyceraldehyde-3-phosphate dehydrogenase (GAPDH)] associated proteins in whole blood and sEVs isolated from both microfluidic device and UC. sEV samples isolated from the microfluidic device and UC showed similar prominent expression of exosomal proteins, while the whole-blood sample exhibited higher expression of GAPDH. Scale bars, 100 μm.

To showcase the utility of the viscoelastic microfluidic device for sEV isolation, we next spiked mNeonGreen-labeled sEVs (mNG-sEVs) into diluted whole blood (γ = 4 : 1). Figure S13 shows the size distribution of the mNG-sEV sample extracted through nanoparticle tracking analysis (NTA), confirming the similarity in size between the mNG-sEVs and the 100-nm-diameter particles. Flow cytometry analysis of the fluid composition at inlet *I*_1_ and outlets *O*_1_, *O*_2_, and *O*_3_ were then performed. Specifically, the relative proportion of cells and sEVs was quantified by gating distinct populations within a two-parameter density plot of violet (405-nm) side scatter (VSSC-H) signal versus fluorescence at 525 nm (FITC-H). As shown in Fig. 3A, three gating regions could be used to discriminate the component subpopulations. First, analysis shows that the initial spiked sample is primarily comprised of WBCs, RBCs, and PLTs (88.6%) with a small proportion (9.7%) of mNG-sEVs (Fig. 3A, inlet *I*_1_). Second, almost all the WBCs and RBCs and a vast majority of PLTs (97.1% in total) are efficiently removed in the cell-depletion module (Fig. 3A, outlet *O*_1_), while lEVs, mEVs, and the remaining blood cells (around 66% in total) are further depleted in the sEV-isolation module via outlet *O*_2_ (Fig. 3A, outlet *O*_2_). Last, mNG-sEVs are extracted from outlet *O*_3_, with a purity and recovery rate above 97 and 87%, respectively (Fig. 3A, outlet *O*_3_). Note that the recovery of mNG-sEVs is actually higher than that of the spiked 100-nm particles. This is likely due to the fact that mNG-labeled sEVs and 100-nm PS particles have quite distinct physical properties that will affect migration (*57*, *58*). Figure 3B shows a comparison of the particle trajectories when experiments are performed in whole blood in both the presence and the absence of the 600-kDa PEO. In the case of blood supplemented with PEO (Fig. 3B, right), the vast majority of WBCs, RBCs, PLTs are efficiently removed in the cell-depletion module, with the remaining components entering the sEV-isolation module, where lEVs and mEVs and the remaining blood components (i.e., WBCs, RBCs, and PLTs) can be collected at outlet *O*_2_.

We then performed detailed downstream analysis of the collected sEVs from outlet *O*_3_, using NTA, atomic force microscopy (AFM), transmission electron microscopy (TEM), and Western blotting (WB). NTA was performed on samples collected from outlets *O*_2_ and *O*_3_ (Fig. 3C) to determine the sEV size distribution and cumulative distribution. It can be seen that sEV concentration is much higher in fluid collected at outlet *O*_3_ than that at outlet *O*_2_, with the cumulative distribution indicating efficient removal of the larger EVs from outlet *O*_2_. From these data, the average size of sEVs in outlets *O*_3_ and *O*_2_ was determined to be 97 nm and 143 nm, respectively. It should be noted that NTA analysis of blood samples at inlet *I*_1_ and outlet *O*_1_ was not performed because the excessively high blood cell concentrations cause blockage of the NTA instrument. That said, flow cytometry analyses of samples at inlet *I*_1_ and outlet *O*_1_ were performed, clearly demonstrating efficient removal of blood cells (Fig. 3A). Figure S14 assesses the impact of PEO on NTA measurements. In contrast to the particle concentrations measured in the microfluidic-derived sEV sample (Fig. 3C), particle concentrations of 0.1% w/v 600-kDa PEO solutions (fig. S14A) are between one and two orders of magnitude lower. Figure S14B presents the particle size distribution of three UC-derived sEV samples in the absence and presence of 0.1% w/v 600-kDa PEO. The particle size distributions remain consistent irrespective of PEO addition. Together, it can be concluded that the presence of PEO has no effect on NTA measurements. We next performed AFM and TEM measurements to observe the morphology of the isolated sEVs collected at outlet *O*_3_. AFM images (fig. S15) depict circular shaped nanostructures with sizes ranging between 50 and 100 nm. However, because AFM imaging cannot distinguish sEVs from non-EV species (such as lipoproteins), TEM measurements were performed. As can be seen in Fig. 3D, characteristic cup-shaped structures (marked by the blue arrow), which are indicative of intact but dehydrated membranous vesicles, are observed. TEM images additionally confirm the presence of lipoproteins within the sample (marked by red arrows), with observations consistent with previous TEM studies of sEV-containing blood samples (*59*, *60*).

Last, WB analysis was used to further validate the efficacy of the microfluidic device in isolating sEVs from whole blood. Specifically, we examined expression levels of EV- and non-EV–associated proteins in three distinct samples: whole blood and sEVs isolated using both the microfluidic device and UC. The total protein concentration of each sample was first measured using a micro bicinchoninic acid (BCA) protein assay, with the same mass of total protein being subsequently loaded in each lane for WB analysis. As shown in Fig. 3E, both sEV samples isolated from the microfluidic device and UC express similar levels of exosomal proteins [heat shock protein 70 (HSP70), tumor susceptibility gene 101 protein (TSG101), cluster of differentiation 81 protein (CD81), and cluster of differentiation 9 protein (CD9)], while extremely low levels of these markers were detected in whole blood. This confirms efficient enrichment of exosomes using both isolation approaches. Significantly, glyceraldehyde-3-phosphate dehydrogenase (GAPDH), which as a “housekeeping” protein is abundantly distributed in cells and widely used as a control for protein normalization in WB analysis, was only detected in the whole-blood sample, being absent in both sEV samples isolated from the microfluidic device and UC. This suggests that the sEV samples were free of cells. Similar results highlighting the absence of GAPDH in sEVs have also been reported previously in other WB studies (*61*–*64*). Together, characterization data confirm that the viscoelastic microfluidic device is capable of passive isolation of sEVs from whole blood with exosomal protein expression levels comparable to those obtained from UC.

### Blood-derived sEVs purified using microfluidics and UC show similar protein composition

**Fig. 4.**
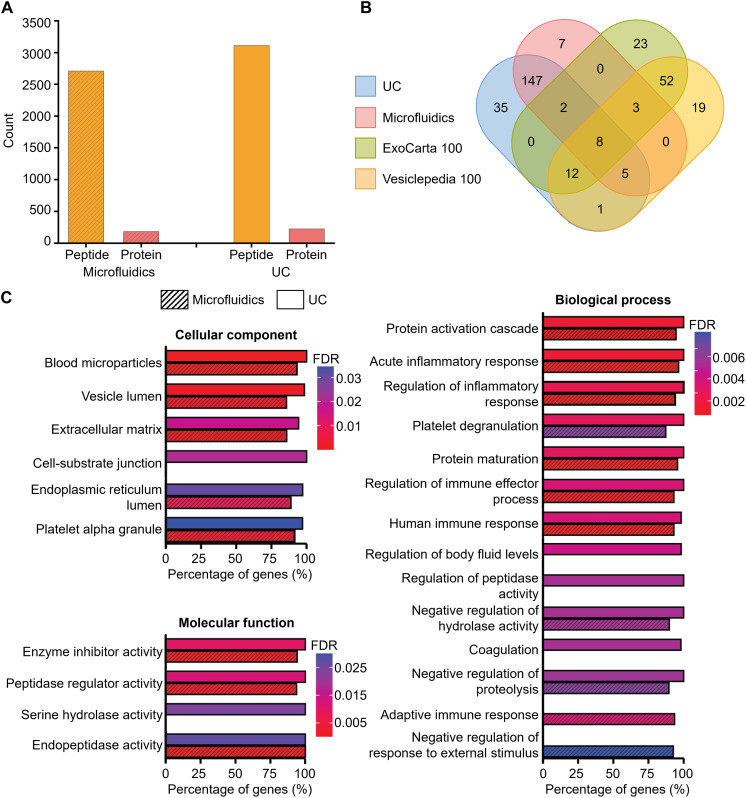
Pathway ORA analysis of EV-associated proteins detected by mass spectrometry. (**A**) Number of peptides and proteins detected from EV samples prepared by microfluidics or UC. (**B**) Overlap between EV-associated proteins isolated by microfluidics or UC, with the top 100 vesicle-enriched proteins from the Vesiclepedia and ExoCarta data repositories. (**C**) Gene ontology–based pathway overrepresentation (ORA) using proteins detected in EVs isolated by the microfluidic device or UC. The Benjamini-Hochberg corrected false discovery rate (FDR) was calculated from a hypergeometric test using all identified proteins as a background.

To further investigate and compare the protein content of blood-derived sEV samples isolated by UC and the microfluidic device, proteins from both sEV samples were extracted and subjected to mass spectrometry–based proteomics analysis using the data-dependent acquisition (DDA) parallel accumulation and serial fragmentation (PASEF) method (*65*). As shown in Fig. 4A, the numbers of detected peptides and proteins were similar for the two sEV isolation methods: 3114 peptides and 210 proteins for UC-derived sEV samples, and 2711 peptides and 172 proteins for microfluidic-derived sEV samples. As shown in Fig. 4B, several of the top 100 EV-associated proteins, reported in available repositories [ExoCarta (*66*) and Vesiclepedia (*67*)], are common to both isolation methods. Gene ontology–based pathway overrepresentation (ORA) showed significant ORA of blood and immune-related processes, as is expected (Fig. 4C and figs. S16 to S18). “Vesicle lumen” and “blood microparticles” were identified as overrepresented cellular components from the detected proteins, thus confirming that EV-associated proteins are overrepresented in both standard UC- and microfluidic-based isolation workflows. To explore whether the two isolation methods exhibit any bias toward specific clusters of blood plasma proteins, in fig. S19, we mapped the 10 proteins exclusively detected in microfluidic-derived samples (but not in UC-derived samples) and the 48 proteins exclusively detected in UC-derived samples (but absent in microfluidic-derived samples) onto a quantitative blood plasma proteome dataset from the Human Protein Atlas (HPA) (*68*). This shows that the nonoverlapping proteins associated with both the microfluidic- and UC-based approaches encompass a comparable palette of blood plasma proteins, indicating no apparent protein enrichment bias with regard to the underlying protein abundance distributions in human blood plasma. To gain more insight regarding the protein contamination from blood plasma, we mapped the 162 overlapping proteins (Fig. 4B) shared between the microfluidic- and UC-derived sEV samples to their corresponding blood plasma abundances from the HPA (fig. S20). Although many of 162 overlapping proteins were abundant in plasma (fig. S20A), the quantitative correlations between their abundance in microfluidics/UC-isolated sEV samples and their blood plasma concentrations from HPA were modest (Spearman regression coefficient, *R* = 0.49 and 0.4, respectively, as shown in fig. S20B, left and middle). In addition, there is a strong correlation (*R* = 0.83) between the microfluidics and UC data, suggesting that protein contamination levels are comparable for both microfluidic- and UC-derived sEV samples (fig. S20B, right). In summary, proteomic analysis confirms that our microfluidic approach exhibits similar performance to the gold-standard UC method in terms of protein composition.

### Evaluation of sEVs collected from HDs and CPs

**Fig. 5.**
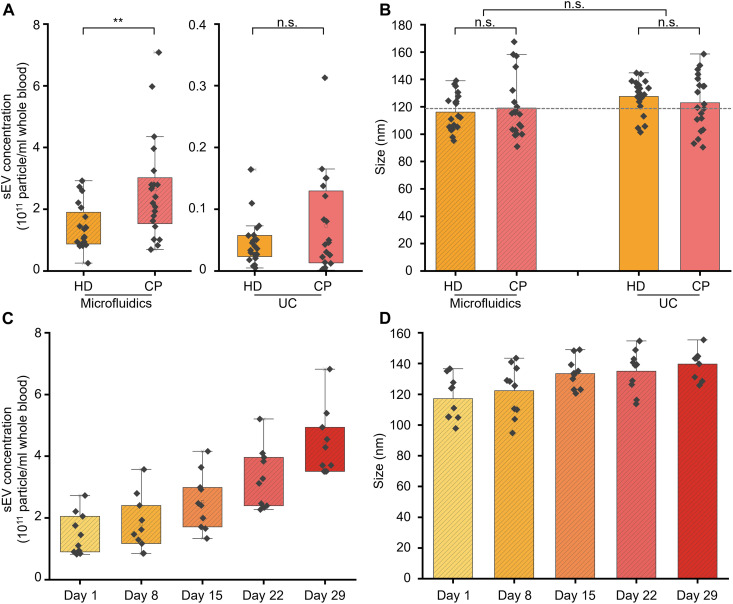
Microfluidic isolation of sEVs in liquid biopsies for cancer diagnosis and monitoring of the concentration and size of sEVs as a function of blood storage time. Comparison of concentrations (**A**) and size distributions (**B**) of sEVs secreted from the blood of 20 HDs and 20 CPs, isolated using the microfluidic device and the UC method. (A) An elevated concentration of secreted sEV can be observed in CPs biopsies compared to that in HDs biopsies. Compared to the UC method, the yield of EVs isolated from the microfluidic device is approximately 30-fold higher (*n* = 20 biological replicates for HDs and *n* = 20 biological replicates for CPs). (B) In terms of average sEV size, there is no significant difference between HDs and CPs samples when using the same isolation method (*n* = 20 biological replicates for HDs and *n* = 20 biological replicates for CPs). sEVs isolated by the microfluidic device have a smaller average size compared to EVs derived from the UC method. Monitoring of sEV concentrations (**C**) and average sizes (**D**) from10 HDs biopsies as a function of blood storage time. sEV concentration is significantly increased over several days of blood storage accompanied by a slight increase in particle size (*n* = 10 biological replicates for HDs). Statistical analysis was performed using one-way analysis of variance (ANOVA) with Tukey’s test for post hoc analysis. **P < 0.01; n.s., *P* > 0.05.

To fully assess the utility of the microfluidic device in isolating sEVs for cancer diagnosis, we measured the concentration and size distribution of isolated sEVs secreted from the blood of 20 HDs and 20 CPs (table S1) and compared the results with the gold-standard UC-based method. As shown in Fig. 5A, in sEV-derived liquid biopsies isolated from both approaches, an elevated concentration of secreted sEV can be observed for CPs when compared to HDs, an observation that is in accordance with previously reported results (*19*, *31*). Figure S21 presents the NTA-based sEV concentration measurements for both HDs and CPs. Significantly, when compared to the UC method, the yield of sEVs isolated from the microfluidic device is approximately 30 times higher. Despite this notable concentration enhancement, it is noted that NTA cannot distinguish between large proteins (such as lipoproteins) and sEVs, and, thus, the higher sEV concentration measured in the microfluidically produced samples may include some large proteins. To investigate this possibility, we performed WB analyses of sEV samples isolated from both the microfluidic device and the UC method, with a view to detecting expression of albumin (a protein contamination indicator). In comparison to sEVs isolated from UC, sEVs isolated from the microfluidic device show a higher level of albumin, indicating a higher level of protein contamination (fig. S22). Accordingly, despite the fact that higher particle concentrations are detected in the samples isolated from microfluidics, it is important to acknowledge the presence of large proteins, such as lipoproteins, that may also be coisolated. In terms of average size, there is no significant difference between sEVs form HDs and CPs, when using the same isolation method (Fig. 5B). However, note that sEVs isolated using the microfluidic device have a marginally smaller average size when compared to sEVs derived from the UC method. This difference can be attributed to the fact that UC uses high centrifugal forces, which can often cause fusion or aggregation of sEVs (*69*). Consequently, these data confirm that the microfluidic device is able to isolate sEVs with high integrity. Moreover, it can be observed that, in the case of UC-based isolation, the coefficient of variations associated with sEV concentration is larger than those associated the microfluidic method (fig. S21). We attribute this observation to inconsistencies in blood sample processing over the course of different days, which causes batch-to-batch variations. Last, note that the microfluidic approach involves minimal human intervention, thereby potentially offering enhanced reproducibility when dealing with clinical cohorts.

### Monitoring sEV variations with blood storage time

Last, we measured the concentration and size of sEVs derived from 10 HD biopsies as a function of blood storage time. As part of the blood aging process, blood cells will shed sEVs, thus increasing the number of blood-borne sEVs over time (*46*, *70*, *71*). Figure 5C confirms such an expectation, demonstrating that sEV concentration is significantly increased over a period of 29 days of blood storage. In addition, data presented in Fig. 5D indicate a more moderate (but measurable) increase in particle size over the same time period. Therefore, these observations confirm the potential of the microfluidic device to additionally monitor blood quality.

## DISCUSSION

We have successfully developed a viscoelastic microfluidic device capable of the continuous and label-free isolation of sEVs from human blood. The device integrates two-unit operations within a monolithic system: a cell-depletion module to remove blood cells (WBCs, RBCs, and PLTs) and an sEV-isolation module to subsequently separate sEVs from lEVs and mEVs. We initially demonstrated the isolation of 100-nm PS particles (sEV mimics) from an aqueous mixture of nanometer- and micrometer-sized particles, with a separation efficiency and recovery of 97 and 77%, respectively. sEV isolation was initially assessed by spiking fluorescent sEVs into whole blood, with the device demonstrating efficient separation of sEVs (97% purity and a recovery rate of 87%) at a sample volumetric flow rate of 200 μl/hour (i.e., whole blood of 40 μl/hour). This level of performance is excellent and compares favorably with the acoustofluidic platform recently reported by Huang and co-workers (*38*) that is able to actively separate sEVs from whole blood with similar purities and recovery rates. In addition, it should be remembered that adoption of a passive separation method is desirable, because application of strong acoustic fields can potentially damage sEV membranes (*72*). While there exist other microfluidic-based techniques for separating sEVs from whole blood (summarized in table S2), our viscoelastic microfluidic device offers numerous advantages, including low operational costs, ease of use, minimal sample preparation requirements, high separation purities, high recovery rates, and excellent size resolution, making it desirable for sEV sorting. All these features are critical considerations when transferring the technology set to a clinical setting. Our clinical studies confirm that the developed microfluidic device is able to separate and quantify sEVs contained within liquid biopsies from both HDs and CPs.

While the microfluidic and UC methods were comparable in terms of measured protein compositions (Figs. 3E and 4), UC requires the use of large, complex instruments, involves the application of centrifugal forces in excess of 110,000*g*, is both time- and cost-intensive, and requires tedious manual interventions. Multiple stage centrifugations typically takes between 5 and 6 hours to complete and are associated with low sEV yields (*12*, *15*, *25*). Compared to UC, the presented microfluidic platform operates in continuous flow, requires a single step to isolate sEVs from 40 μl of whole blood, takes less than 1 hour, results in a 30-fold higher yield based on NTA measurements. However, note that NTA lacks the ability to differentiate between large plasma proteins and sEVs. As a result, the elevated sEV yields observed for microfluidic-derived samples may be partially attributed to the presence of these large proteins. From a clinical perspective, the microfluidic method is capable of processing low volume blood samples, is highly reproducible (due to the lack of manual interventions), and ensures high sEV isolation yields. In addition, the proposed microfluidic chip comprises a single glass side and no more than a few grams of silicone polymer, with a total cost of less than 1 USD per chip. All these features suggest important utility in both medical diagnosis and treatment.

Last, note that, in future, analytical throughput may be increased through multiplexing. Tay *et al.* (*31*) and Nam *et al.* (*32*) have leveraged parallel design strategies for both inertial and viscoelastic methods to separate sEVs from whole blood using volumetric flow rates at 4.8 and 2.4 ml/hour, respectively. Although the current microfluidic platform operates at lower volumetric flow rates, throughput can be increased through parallelization and without increasing the complexity of the device operation, as shown in fig. S23. Moreover, we expect that the transfer of the microfluidic platform from a laboratory prototype to a mass-produced diagnostic tool will be straightforward and likely involve well-established methods for fabricating thermoplastic devices (*73*, *74*). In addition, the current platform can be integrated with supplementary downstream analyses, such as surface plasmon resonance-based assays, to allow for disease-specific sEV detection (*75*, *76*). Here, we have developed a simple microfluidic approach to passively isolate sEVs from whole blood, achieving excellent sEV recovery and purity. Our approach offers several benefits including low-cost of manufacture, ease of operation, and minimal sample preparation. We envision that the presented platform will become a versatile tool for sEV-based biological investigations and clinical application by simplifying the isolation of sEVs from complex biofluids.

## MATERIALS AND METHODS

### Device design and fabrication

Microfluidic devices were fabricated using standard soft lithographic techniques. First, microchannel patterns were designed in AutoCAD 2018 (Autodesk, San Rafael, USA) and printed onto a high-resolution film photomask (Micro Lithography Services, Chelmsford, UK). A 55-μm-thick resist layer (SU-8 3050, MicroChem, Westborough, USA) was coated onto a silicon wafer. The photomask was then used to pattern master structures onto the SU-8–coated silicon wafer by ultraviolet (UV) exposure. Next, the master structures were developed for 3 min using 1-methoxy-2-propyl acetate (Sigma-Aldrich, Buchs, Switzerland). After fabrication, the SU-8 master mold was exposed to chlorotrimethylsilane (Sigma-Aldrich, Buchs, Switzerland) vapor for at least 5 hours in a desiccator at 150 mbar to aid subsequent removal of polydimethylsiloxane (PDMS). A 10:1 (wt/wt) mixture of PDMS monomer to curing agent (Sylgard 184, Dow Corning, Midland, USA) was degassed in a desiccator for 60 min, poured over the master mold, cured at 70°C for 4 hours, and then peeled off the master mold. A 22-gauge puncher (I and Peter Gonano, Niederösterreich, Austria) was used to create inlet and outlet ports in the structured PDMS substrate. Last, the microfluidic device and a 24 mm–by–75 mm–by–1 mm glass slide (Menzel-Glaser, Braunschweig, Germany) were treated in a Zepto air plasma chamber (Diener Electronic, Ebhausen, Germany) for 1 min and contacted. The entire assembly was then placed on a hot plate at 120°C for 2 hours to strengthen the bonding of the PDMS substrate to the glass slide.

### Viscoelastic fluid preparation and characterization

Viscoelastic stock solutions were prepared by fully dissolving 600-kDa polyethylene oxide (PEO, Sigma-Aldrich, Buchs, Switzerland) in 1× PBS buffer (Thermo Fisher Scientific, Reinach, Switzerland) to a concentration of 1% (w/v). Stock solutions were aged at room temperature for 1 week to ensure a uniform viscosity. Before each experiment, solutions of the desired PEO concentration were prepared by diluting the 1% stock PEO solution with 1× PBS. Viscosities of all fluids (fig. S3) were measured at room temperature using a MCR 502 compact rheometer equipped with a double gap (DG 26.7) tool (Anton Paar, Ostfildern, Germany).

### Flow cytometry

Flow cytometry measurements were conducted using a CytoFLEX flow cytometer (Beckman Coulter, Pasadena, USA). To evaluate PS particle separation performance, violet (405-nm) side scatter (VSSC-H) signal versus fluorescence signal emitted at 585 nm (PE-H) was used for gating. For mNG-sEV separation from blood, a plot of VSSC-H signal versus fluorescence signal emitted at 525 nm (FITC-H) was adopted. For all measurements, the sample flow rate was set to 30 μl/min. Data were acquired using the CytExpert software (Beckman Coulter, Pasadena, USA). The results were later processed using the FlowJo software (FlowJo, Ashland, USA). Figure S24 describes the gating strategy used in flow cytometry experiments. PBS, a mNG-sEV sample, and whole-blood sample were analyzed separately. WBCs, RBCs, PLTS, lEVs, and mEVs were gated based on FSC-H and VSSC-H values. mNG-sEVs were gated on the basis of FITC-A and VSSC-H values.

### PS sample preparation

PS particles having average diameters of 12, 7, and 3 μm (Sigma-Aldrich, Buchs, Switzerland) and fluorescent PS particles having average diameters of 1 μm, 500 nm, and 100 nm (Invitrogen, Carlsbad, USA) were dispersed in PBS buffer and in whole blood supplemented with the PEO solution.

### Device operation and data acquisition

Sample and guide fluids were loaded into 1-ml Primo syringes (Codan Medical ApS, Rødby, Denmark) and 5-ml Hamilton syringes (Hamilton Laboratory Products, Reno, USA), respectively, and delivered into the microfluidic device using neMESYS precision syringe pumps (CETONI, Korbussen, Germany). The microfluidic device was mounted on an Eclipse TS100 inverted microscope (Nikon, Zürich, Switzerland) equipped with a EOSens 3CL high-speed camera (Mikrotron, Unterschleissheim, Germany) for bright-field imaging and a CoolSNAP HQ2 charge-coupled device (CCD) camera (Roper Scientific, Ottobrunn, Germany) for fluorescence imaging. Bright-field images were acquired using a MCWHD3 white light LED (Thorlabs, Dachau, Germany) in combination with a 20×, 0.45 numerical aperture (NA) S Plan Fluor objective (Nikon, Zürich, Switzerland) and a 0.7× demagnification lens (yielding a total magnification of 14×). Fluorescence measurements were conducted using an Intensilight C-HGFI mercury light source (Nikon, Zürich, Switzerland) as an excitation source combined with tetramethylrhodamine filter set (AHF Analysentechnik, München, Germany), with emission being collected with a 10×, 0.3 NA Plan Fluor objective (Nikon, Zürich, Switzerland). Images were then processed using ImageJ (U.S. National Institutes of Health, Bethesda, USA).

### TEM imaging

Four hundred mesh carbon-coated grids (Quantifoil Micro Tools, Jena, Germany) were freshly glow discharged in a PELCO easiGlow system (Ted Pella, Redding, USA) for 30 s at 25 mA. Immediately afterward, 4 μl of an sEV sample was placed on the grid and left for 1 min. Subsequently, excess liquid was removed using filter paper and the grid washed twice with MilliQ water (Merck, Schaffhausen, Switzerland). Next, the grids were negatively stained using two consecutive drops of 1% uranyl acetate for 1.5 min each. After thorough air drying, the grid was imaged using a CCD KeeneView camera within a Morgagni 268 TEM (Thermo Fisher Scientific, Reinach, Switzerland) operating in bright-field mode at 100 kV.

### AFM imaging

Substrates for AFM imaging were prepared by pipetting a 5-μl sample of sEVs onto a 5 mm–by–5 mm silicon wafer. The sample was allowed to adsorb to the silicon wafer for 10 min, followed by air drying. Subsequently, substrates were washed with MilliQ water (Merck, Schaffhausen, Switzerland) and dried with nitrogen gas. AFM images were obtained at room temperature using a Cypher AFM (Asylum Research, Santa Barbara, USA) operating in nontapping mode, with the acquired images being processed using Gwyddion software (version 2.25).

### Preparation of mNG-sEVs

Human embryonic kidney (HEK) 293T flp-in TREX cells (R78007, Thermo Fisher Scientific, Zurich, Switzerland) expressing mNG-HRAS (FSN-HRAS) were conditioned to grow in Pro293 (Lonza, Visp, Switzerland), a serum-free medium for 293 adherent cells supplemented with 1% FBS medium (Thermo Fisher Scientific, Zurich, Switzerland), penicillin (100 U/ml; Sigma-Aldrich, Buchs, Switzerland), and streptomycin (100 μg/ml; Sigma-Aldrich, Buchs, Switzerland). Cells were then detached from the culture vessel using Accutase (STEMCELL Technologies, Köln, Germany).

To obtain conditioned culture medium (CCM) containing mNG-sEVs, 2 × 10^7^ HEK293T FSN-HRAS cells were seeded in two, 15-cm-diameter, dishes with Pro293a medium supplemented with penicillin (100 U/ml) and streptomycin (100 μg/ml) but without FBS medium. The next day, doxycycline (2 μg/ml; Sigma-Aldrich, Buchs, Switzerland) was added to induce protein expression. After 3 days of cell growth, CCM from the two dishes was collected in a 50-ml Falcon tube and centrifuged for 5 min at 500*g* to remove the cell debris. The supernatant was then centrifuged for an additional 5 min at 1000*g* to remove other large bioparticles. Afterward, the supernatant was serially filtered through 0.8-μm and 0.2-μm syringe filters (Corning, Bodenheim, Germany). Last, a Centricon 70 Plus centrifugal filter (Sigma-Aldrich, Buchs, Switzerland) was used to concentrate the CCM down to 500 μl as per the manufacturer’s instructions. More detailed procedures regarding the mNG-sEV preparation can be found in our previous publication (*29*).

### Separation of sEVs from whole blood via UC

The purification of sEVs using UC was performed as follows. EDTA anticoagulated blood samples from either CPs or HDs were centrifuged twice, at 2500*g* for 15 min, to obtain PLT-free plasma supernatant. Next, the obtained plasma was suspended with an equal volume of 1× PBS and centrifuged at 2000*g* for 30 min. The collected supernatants were then centrifuged at 110,000*g* for 2 hours at 4°C using an OptimaTM Max-xp Ultra Centrifuge (Beckman Coulter, Brea, USA) to pellet the sEVs. The purified sEV pellets were collected, resuspended with 1× PBS, and centrifuged again at 110,000*g* for 2 hours at 4°C before collection.

### WB analysis

Examination of EV and non-EV protein markers was achieved through WB analysis. Briefly, in diluted whole blood, an sEV sample from the microfluidic device and a diluted sEV sample produced by UC were mixed with radioimmunoprecipitation assay (RIPA) lysis buffer (Sigma-Aldrich, Buchs, Switzerland) and added to 1× cOmplete EDTA-free protease inhibitor cocktail (Roche Diagnostics, Manheim, Germany). The volume ratio between the sEV sample from microfluidic device and the RIPA lysis buffer was 10:1. The mixture was lysed on ice for 5 min. Next, samples were centrifuged at 14,000*g* for 15 min at 4°C, and supernatants were collected. The relative protein concentration for each sample was then measured using the MicroBCA Protein Assay Kit (Thermo Fisher Scientific, Reinach, Switzerland) following the manufacturer’s instructions. Subsequently, 4× Laemmli sample buffer (Bio-Rad Laboratories, Hercules, USA) supplemented with 10% v/v 2-mercaptoethanol (Sigma Aldrich, Buchs, Switzerland) was added to the samples to a concentration of 1× Laemmli, and, then, the mixtures were heated to 95°C for 5 min. Samples (containing identical amounts of protein) were then loaded on Mini-Protein TGX Stain-free Precast Gels (Bio-Rad Laboratories, Hercules, USA). Gels were placed in a tank filled with tris/glycine/SDS running buffer (Bio-Rad Laboratories, Hercules, USA) and electrophoresed for approximately 20 min at 200 V. Next, the stain-free gels were UV-activated using the ChemiDoc MP imaging system (Bio-Rad Laboratories, Hercules, USA), and proteins were transferred from the gel onto a suitable membrane for antibody staining and detection. Specifically, proteins were transferred to polyvinylidene difluoride membranes (Bio-Rad Laboratories, Hercules, USA) using the Trans-Blot Turbo Transfer System (Bio-Rad Laboratories, Hercules, USA) running the “high molecular weight” program. Membranes were then blocked in an EveryBlot Blocking buffer (Bio-Rad Laboratories, Hercules, USA) for 5 min and incubated overnight at 4°C with primary antibodies diluted in the blocking buffer. Antibodies and their concentrations are as follows: anti-HSP70 (1:1000; ab181606, Abcam, Cambridge, UK), anti-TSG101 (1:2000; ab125011, Abcam, Cambridge, UK), anti-CD9 (1:1000; ab263019, Abcam, Cambridge, UK), anti-CD81 (1:2000; ab109201, Abcam, Cambridge, UK), GAPDH (1:10,000; ab181602, Abcam, Cambridge, UK), and albumin (1:2000; P0356, Dako, Glostrup, Denmark). Next, each membrane was washed five times (5 min each time) with tris-buffered saline containing 0.1% Tween 20 (TBS-T). All membranes apart the one carrying the albumin were incubated with the secondary horseradish peroxidase (HRP)–conjugated antibody (ab205718, Abcam, Cambridge, UK) diluted with buffer at a ratio of 1:10,000, for 1 hour at room temperature. In the case of albumin, the primary antibody is peroxidase-conjugated and thus can be directly imaged after washing. Accordingly, the membrane did not require incubation with a secondary antibody for detection. After incubation, membranes were washed five times (5 min each time) with TBS-T buffer, incubated with Clarity Western ECL Substrate (Bio-Rad Laboratories, Hercules, USA) for 5 min, and lastly imaged on a ChemiDoc system (Bio-Rad Laboratories, Hercules, USA).

### Nanoparticle tracking analysis

Size distribution and concentration analyses of sEVs were performed using a ZetaView PMX 120-Z NTA instrument (Particle Metrix, Meerbusch, Germany) equipped with a CMOS camera and a 405-nm laser. Samples were diluted in filtered 1× PBS buffer to a concentration between 10^7^ and 10^8^ particles/ml and then injected into the instrument using a 1-ml syringe. Eleven positions with two readings per position were recorded for each sample. Data were analyzed using ZetaView software (Particle Metrix, Meerbusch, Germany), with hydrodynamic diameters of multiple particles being calculated via the Stokes-Einstein equation. The instrument’s default post-acquisition parameter settings with the 5-nm width of the bin class were applied.

### Numerical simulations

For all simulations, we assumed that the presence of particles does not significantly affect the flow field. Accordingly, particle trajectories and flow fields were solved independently. Moreover, and on the basis of prior reports, if diluted PEO solutions have constant viscosities over a wide range of shear rates, then the velocity profile for a steady flow will be Newtonian (*30*). In our case, and as shown in fig. S3, viscosities are independent of shear rates at all PEO concentrations used. Therefore, a two-dimensional impressible Newtonian model was used to calculate flow fields using COMSOL Multiphysics 5.5 (COMSOL, Burlington, USA). The flow field distribution was calculated on the basis of

$$\rho_{f}\nabla \cdot u=0$$ (1)

$$\rho_{f}(u\cdot \nabla )u=-\nabla p+F$$ (2)

where ρ_f_ is the fluid density (1000 kg m^−3^), ***u*** is the fluid velocity, *p* is the pressure, ***F*** = η∇***u***^2^ is the volume force vector, and η is the dynamic viscosity of the fluid. In the current study, different concentrations of PEO solutions were used. On the basis of the gyration radius of a 600-kDa PEO molecule, 48 nm, the diffusion coefficient *D*_0_ was estimated to be 4.45 × 10^−12^ m^2^ s^−1^ (*55*, *77*). The Péclet number (*Pe*) is given by

$$Pe=u_{\max}w/D_{0}$$ (3)

For a 20-μm-wide microfluidic channel and a maximum velocity of 1.84 ms^−1^, this yields a *Pe* of 8.27 × 10^6^, indicating that PEO diffusion is negligible with respect to advection (*55*). After performing flow simulations with COMSOL, data were imported into MATLAB using the COMSOL LiveLink toolbox.

On the basis of Newton’s second law, the force balance equation can be written as

$$\sum F=\rho_{p}V_{p}a_{p}$$ (4)

where ρ_p_ is the particle density, *V*_p_ is the particle volume, and ***a***_p_ is the particle acceleration. Particles immersed in a non-Newtonian fluid will be acted upon by elastic forces (***F***_e_), viscous drag forces (***F***_d_), inertial lift forces (***F***_i_), virtual mass forces (***F***_v_) arising from acceleration differences between particles and the fluid, and buoyancy forces (***F***_b_), i.e.

$$F_{e}=C_{e}a^{3}\nabla N_{1}$$ (5)

$$F_{d}=3\pi a\eta (u-v_{P})$$ (6)

$$F_{i}=C_{i}\rho_{f}u_{\max}^{2}a^{4}/w^{2}$$ (7)

$$F_{v}=\frac{1}{2}\rho_{f}V_{p}(u-v_{P})$$ (8)

$$F_{b}=(\rho_{p}-\rho_{f})V_{p}g$$ (9)

Here, *C*_e_ is the elastic lift coefficient, *a* is the particle diameter, *N*_1_ is the first normal stress difference, ***v****_P_* is the velocity of the particle, *C*_i_ is the inertial lift coefficient, and *g* is gravitational acceleration. *N*_1_ is calculated through $N_{1}=2\mu_{p}\lambda {\dot{\gamma }}^{2}$ using the Oldroyd-B model (*78*). where μ_p_ is the polymeric contribution to the viscosity, λ is the relaxation time, and $\dot{\gamma }$ is the shear rate obtained from COMSOL simulation. Here, we set μ_p_ = 0.41 mPa · s, λ = 0.55 ms and *C*_e_ = 0.0184, according to previous reports (*30*). To evaluate inertial effects on particle migration, the channel Reynolds number (*R*) and particle Reynolds number (*R*_P_) were calculated using experimentally determined volumetric flow rates, i.e.

$$R=\rho u_{\max}w/\eta$$ (10)

$$R_{P}=R{\left(a/w\right)}^{2}$$ (11)

where ρ = 1000 kg m^−3^ and η = 1.43 mPa · s (the average viscosity of 0.1 and 0.15% w/v 600-kDa PEO solutions). The calculated values were *R* = 25, *R*_P_ = 0.56, 0.063, 0.0157, and 0.006 for 3-μm, 1-μm, 500-nm, and 100-nm particles, respectively. These values indicate that inertial effects for *R*_P_ are negligible. Accordingly, ***F***_i_ can be neglected due to the small particle Reynolds numbers, and, additionally, for neutrally buoyant particles, ***F***_b_ can also be neglected. Therefore, by inserting Eqs. 5, 6, and 8 into Eq. 4 and solving against *a*_p_, the following equation can be derived

$$a_{p}=\frac{dv_{p}}{dt}=\frac{\sum F}{\rho_{p}V_{p}}=\frac{C_{e}a^{3}\nabla N_{1}+3\pi a\eta (u-v_{P})+\frac{1}{2}\rho_{f}V_{p}(u-v_{P})}{\rho_{p}V_{p}}$$ (12)

Subsequently, a fourth-order Runga-Kutta technique was used to solve the differential equation. A description of the detailed particle tracking algorithm can be found elsewhere (*79*).

### Proteomics analysis

sEV samples isolated from both the microfluidic device and UC were first concentrated using the qEV Concentration Kit (Izon Science, Christchurch, New Zealand). Specifically, sEV samples were incubated with Nanotrap EV Particles (NEVPs; Izon Science, Christchurch, New Zealand) at room temperature for 2 hours and then centrifuged at 10,000*g* for 10 min to form pellets. The sEV-NEVP pellets were then lysed, and the released proteins were ultrasonicated and denatured in tris-HCl buffer (pH 7.8) supplemented with 50 μl of 4% SDS at 95°C for 5 min. Subsequently, samples were placed in an ice-cold sonication bath six times for 30 s, with 30-s intervals on ice to reduce overheating. NEVPs were then removed by centrifugation at 10,000*g* for 10 min. The supernatants were then harvested, followed by performance of a BCA assay to determine protein concentrations. Trichloroacetic acid precipitation was then used to remove contaminants including any residual SDS. Next, proteins were digested with trypsin. Subsequently, the peptides were injected into a timsTOF Pro mass spectrometer (Bruker Daltonics, Bremen, Germany) coupled to an Evosep One high-throughput chromatography system (Evosep, Odense, Denmark) using a 44-min gradient. Samples were acquired in ddaPASEF mode with a mass range of 100 to 1700 mass/charge ratio, accumulation and ramp times of 100 ms, and an ion mobility range of 0.6 to 1.6 Vs/cm^2^. Raw data were processed and quantified using the Fragpipe/MSFragger (version 3.4 with default settings) (*80*). The resulting protein matrix (combined_protein.tsv) was imported into RStudio (version 2022.07.1) for downstream analysis. ORA analysis was performed using the WebgestaltR package (https://github.com/bzhanglab/WebGestaltR). All quantified proteins (*n* = 220) were used as a background for ORA. The gene set size was set to 25 to 500, and the false discovery rate cutoff was set to 0.05. The top 100 vesicle-associated proteins were downloaded from the Vesiclepedia (http://microvesicles.org/index.html) and the Exocarta (www.exocarta.org) data repositories. The human plasma proteome dataset was downloaded from HPA (www.proteinatlas.org/humanproteome/blood+protein/proteins+detected+in+ms).

### Human blood samples

All blood samples were collected in EDTA tubes. CP samples (20 in total) were obtained from the University Hospital Zurich (Zurich, Switzerland). Written informed consent was obtained and documented for all patients, with samples being deidentified for patient confidentiality under the “Generalkonsent des USZ” of the University Hospital Zurich. Blood samples from HDs (20 in total) were obtained from blood donation center, Blutspende Zürich (Schlieren, Switzerland). The study was conducted in accordance with the principles of the Declaration of Helsinki, and the project was approved by the Swiss Association of Research Ethics Committees (BASEC-Nr: 2019-01721). Information regarding age, gender, and cancer type can be found in table S1.

### Statistical analysis

Statistical analysis was carried out using OriginPro 2021b software (OriginLab, Northampton, USA). Unless otherwise stated, one-way analysis of variance (ANOVA) was performed with Tukey’s test for post hoc analysis. *P* values of >0.05 were considered not significant. Of note, ** indicates *P* values less than 0.01; n.s. is not significant.

## Supplementary Materials

### This PDF file includes:

Supplementary Texts S1 and S2 Figs. S1 to S24 Tables S1 and S2
