# Supplementary material for: Direct isolation of small extracellular vesicles from human blood using viscoelastic microfluidics
Source: Sci Adv. 2023 Oct 6;9(40):eadi5296. doi: 10.1126/sciadv.adi5296 (PMC10558121; doi:10.1126/sciadv.adi5296)
Supplement: Supplementary file 1 — Supplementary Texts S1 and S2 Figs. S1 to S24 Tables S1 and S2 [file sciadv.adi5296_sm.pdf]

Science Advances
AAAS

Supplementary Materials for

Direct isolation of small extracellular vesicles from human blood using viscoelastic microfluidics

Yingchao Meng *et al.*

Corresponding author: Andrew J. deMello, andrew.demello@chem.ethz.ch; Stavros Stavrakis, stavros.stavrakis@chem.ethz.ch

Sci. Adv. **9**, eadi5296 (2023)
DOI: 10.1126/sciadv.adi5296

This PDF file includes:

Supplementary Texts S1 and S2
Figs. S1 to S24
Tables S1 and S2

Supplementary Text 1

Influence of other geometrical parameters, guide flow rates and sample flow rates on separation performance.

Fig. S5, **S6** and **S7** further assess the influence of other geometrical parameters, guide flow rates and sample flow rates on separation performance. **Fig. S5** presents particle trajectories and flow field distributions for different geometrical parameters. The geometrical parameters used in the previous simulation (**Fig. 2**) are shown in **fig. S1**. As shown in **fig. S5A**, **B**, **C** and **D**, altering the channel width to 25 μm (from 20 um) leads to a greater particle deviation from the centerline. Under these conditions, 3 μm diameter particles cannot be separated in the cell-depletion module (**fig. S5A** and **B**). **Fig. S5E**, **F**, **G** and **H** further show the effect of the length of the serpentine channel, situated at the end of the cell depletion module, on particle separation. Modifying the length of the serpentine channel to 8 cm reduces flow resistance in this segment, guiding 1 μm particles towards outlet O_1. This outcome is not preferable since 1 μm particles should be separated in the sEV-isolation module. **Fig. S6** and **S7** show particle trajectories and flow field distributions for varying guide and sample flow rates, respectively. In **fig. S6**, the sample flow rate at I_1, remained constant at 200 μL/h, while the total guide flow rate (at I_2 and I_3) was set at 5000 μL/h. When the guide flow rates were set to 1000 μL/h and 4000 μL/h, respectively, particles are still directed to the desired outlets (**fig. S6A**, **B**, **C** and **D**). However, in the cell-depletion module, the suboptimal focusing of 3 μm diameter particles could potentially introduce contamination in the subsequent sEV-isolation module. It is also worth noting that due to the relatively low sample-to-guide flow rate ratio of 1:5, initial particle positions were not aligned closely to the wall. **Fig. S6E**, **F**, **G** and **H** highlight a scenario where the guide flow rates at I_2 and I_3 were set to 4000 μL/h and 1000 μL/h, respectively. In this case, both 3 μm and 1 μm particles are directed towards outlet O_1 within the cell-depletion module, while 500 nm particles fail to exit through the desired outlets. In **fig. S7**, the guide flow rates at I_2 and I_3 were set to 2000 μL/h and 3000 μL/h, respectively. With the sample flow rate at I_1 set to 50 μL/h, **fig. S7A**, **B**, **C** and **D** demonstrate particle separation performance comparable to that shown in **Fig. 2**. However, the sample flow rate at I_1(200 μL/h) in **Fig. 2** is higher. Adjustment of the sample flow rate at I_1 to 400 μL/h still allows for effective separation of particles of different sizes with a guide flow rate of 2000 μL/h at I_2 and a guide flow rate of 3000 μL/h at I_3 (**fig. S7E**, **F**, **G** and **H**). This is predicted by simulation, assuming that particles originate at the same point (**fig. S7E**). However, in "real world" experiments, initial particle positions are not fixed, and a low sample-to-guide flow rate ratio of 1:5 is likely to significantly impact particle separation efficiency.

Supplementary Text 2

Definitions of “purity”, “recovery” and “separation efficiency”

Purity = (number of desired species collected in the target outlet divided by the total number of species collected in the target outlet) × 100

Recovery = (number of desired species collected in the target outlet divided by the number of desired species introduced through inlet I_1) × 100

Separation efficiency = (number of desired species collected in the target outlet after normalization divided by the total number of species collected in the target outlet after normalization) × 100

To determine the separation efficiency, the purities of particles with sizes of 3 μm, 1 μm, 500 nm, and 100 nm at inlet I_1 are normalized to 33.33%, 16.67%, 16.67%, and 33.33%, respectively.

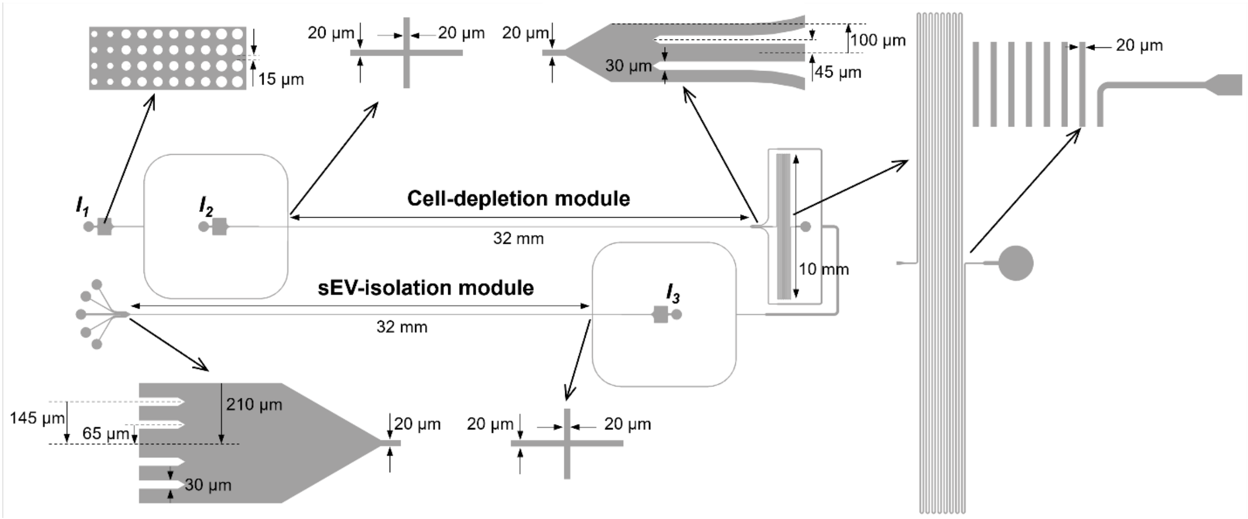

Fig. S1. CAD design of the microfluidic device (55 µm feature height) used for isolation of sEVs from human blood. The microfluidic device (middle) consists of two modules: a cell-depletion module (top right) and an sEV-isolation module (bottom left). Both consist of a 32 mm long and 20 µm wide channel for particle separation. At the end of the cell depletion module, there is a 16 cm long, 20 µm wide serpentine channel to regulate flow resistance. The sample fluid is injected through inlet I_1, and guide fluids flow through inlet I_2 and I_3.

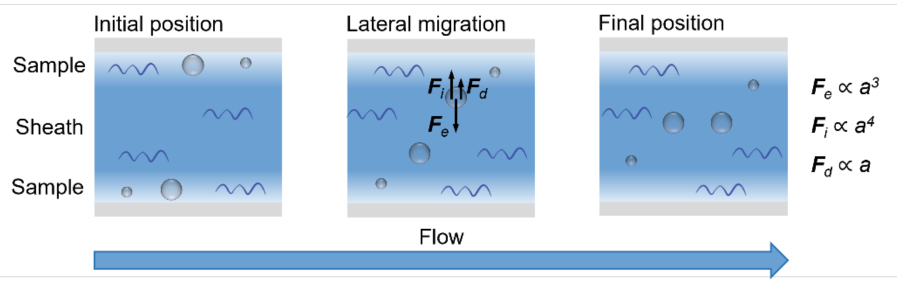

Fig. S2. Particle separation mechanism. Sample flow is initially aligned close to the sidewalls using a central guide flow. Particles immersed in the viscoelastic medium primarily experience three size-dependent forces: $\boldsymbol{F}_e \propto a^3$, $\boldsymbol{F}_d \propto a^4$ and $\boldsymbol{F}_i \propto a$. Competition among these forces results in distinct lateral migration trajectories for different-sized particles.

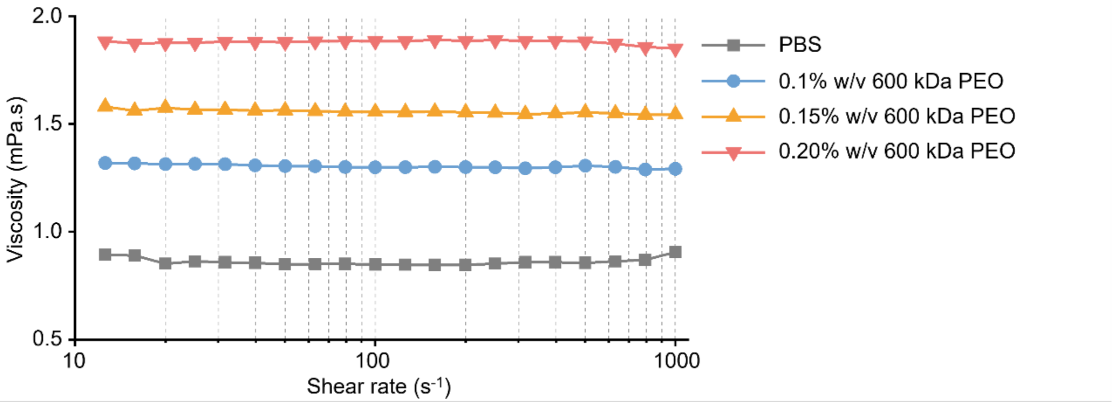

Fig. S3. Viscosity values as a function of shear rate measured for different concentration 600 kDa PEO solutions. Measurements were conducted at room temperature and at shear rates between 10 and 1000 s^{-1} using a rotational rheometer. All PEO solutions show an essentially constant viscosity over the measured range of shear rates.

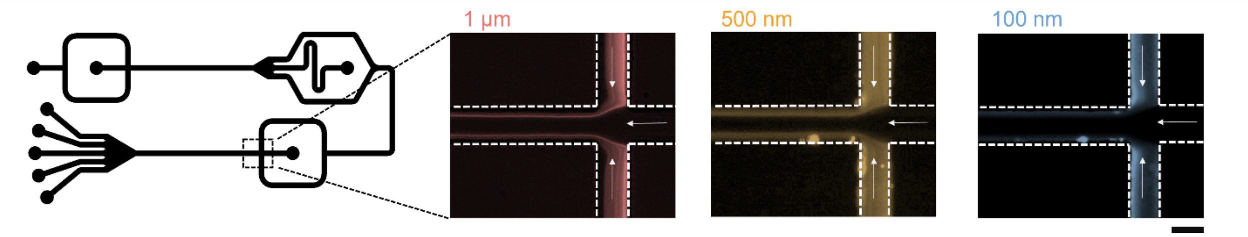

Fig. S4. Particle trajectories at the inlet of the sEV-isolation module. 1 μm, 500 nm, and 100 nm beads enter the sEV-isolation module at different locations. The scale bar is 20 μm.

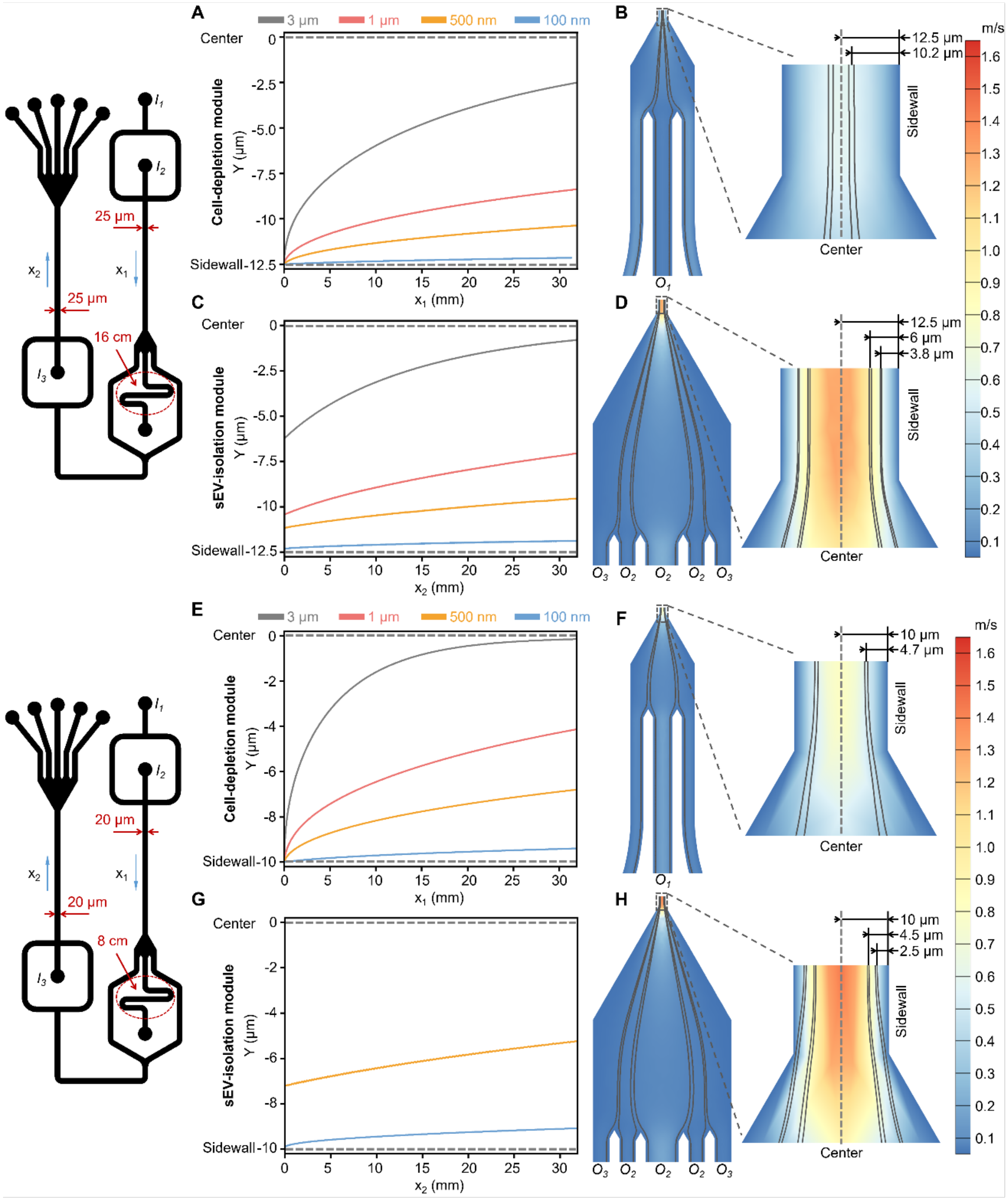

Fig. S5. Particle trajectories and flow field distributions under different geometrical parameters. In (**A**), (**B**), (**C**) and (**D**), both channel widths of the straight microchannels featured in the cell-depletion and the sEV-isolation modules were set to 25 µm. In (**E**), (**F**), (**G**) and (**H**), the length of the serpentine channel located at the end of the cell depletion module was set to 8 cm. For both simulations, flow rates for inlets I_1, I_2 and I_3 were set to 200 µL/h, 2000 µL/h and 3000 µL/h.

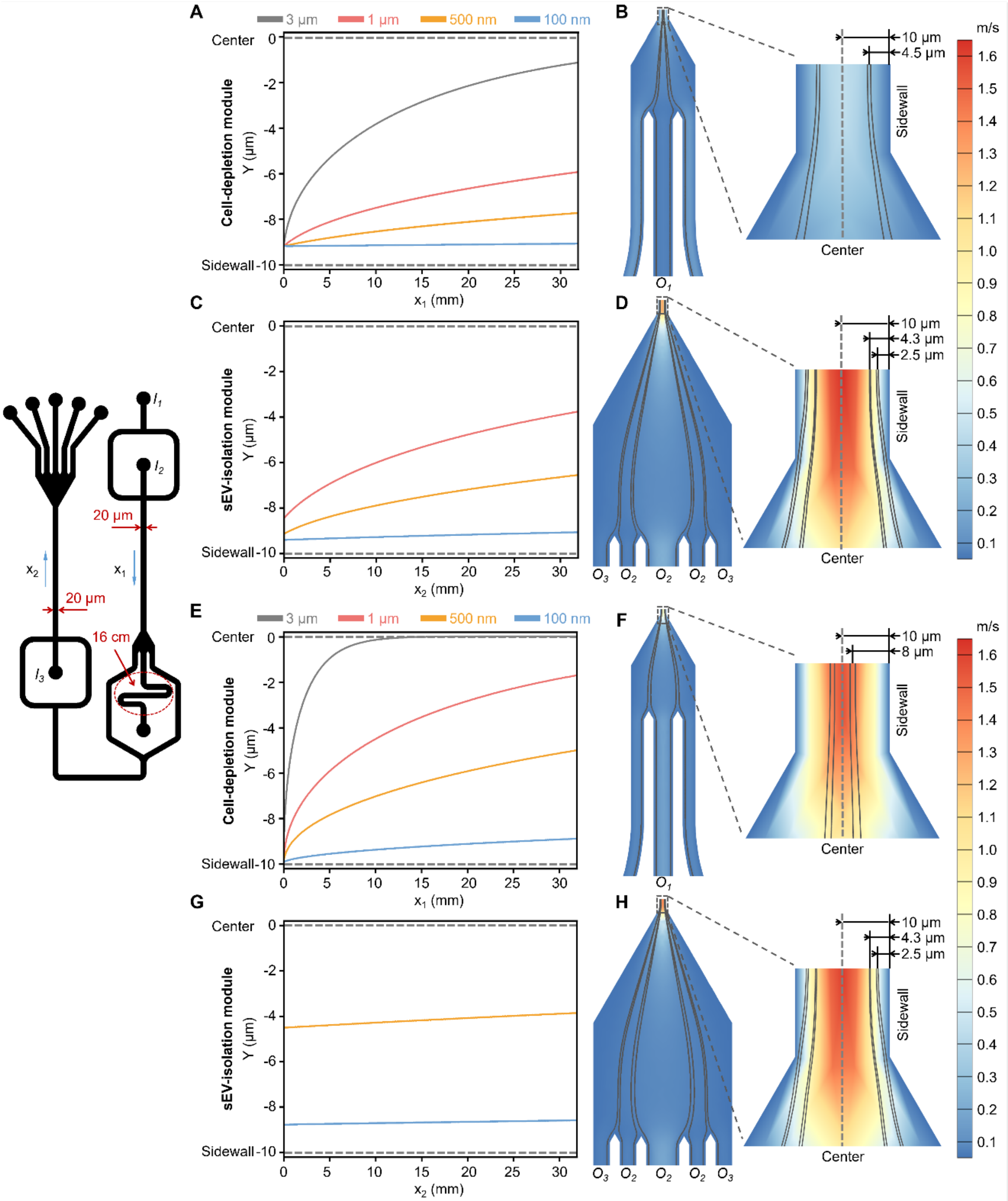

Fig. S6. Particle trajectories and flow field distributions under different guide flow rates at I_2 and I_3. In (**A**), (**B**), (**C**) and (**D**), the guide flow rates at I_2 and I_3 were set to 1000 μL/h and 4000 μL/h, respectively. In (**E**), (**F**), (**G**) and (**H**), the guide flow rates at I_2 and I_3 were set to 4000 μL/h and 1000 μL/h, respectively. For both simulations, the sample flow rate at I_1 was fixed at 200 μL/h.

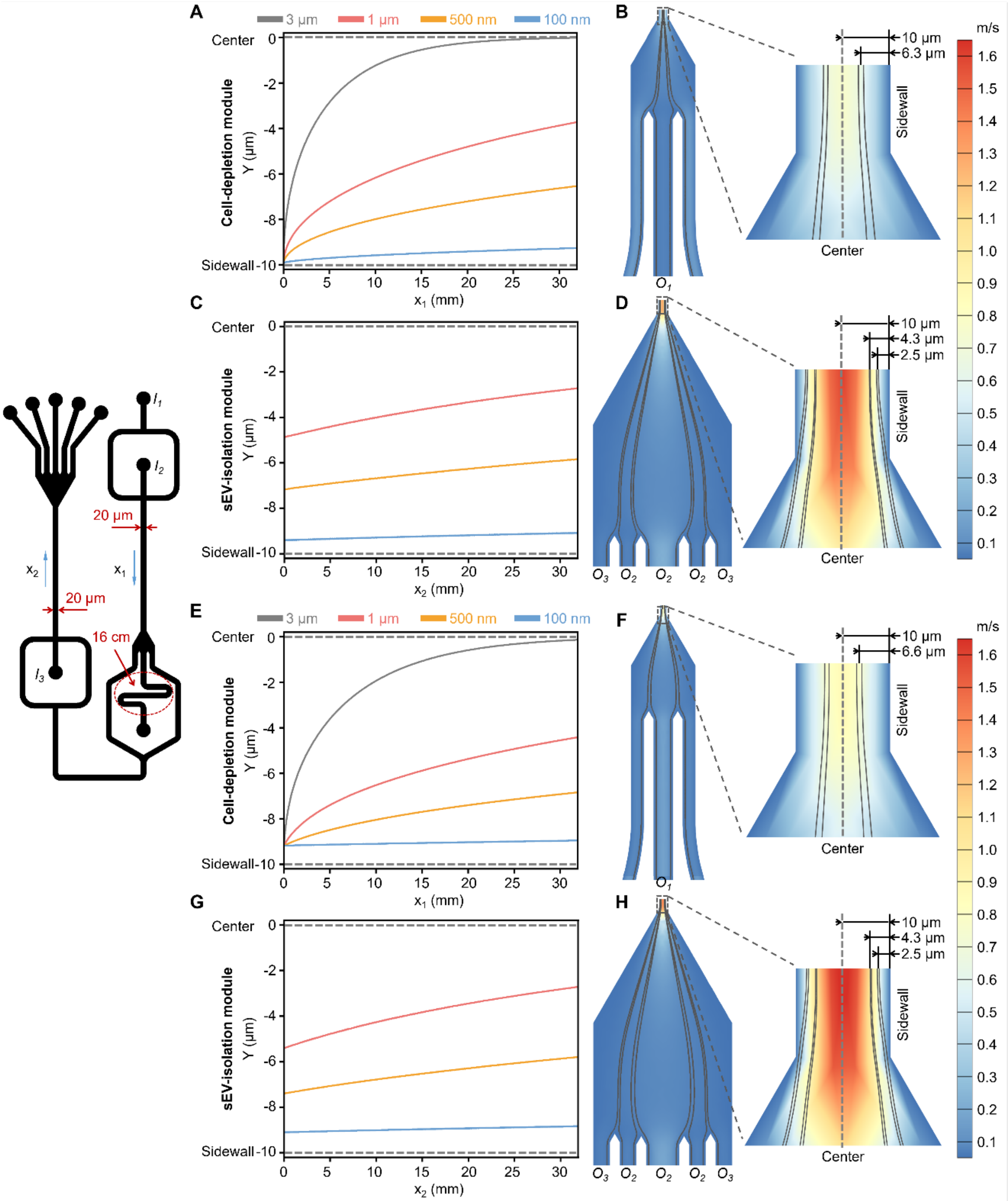

Fig. S7. Particle trajectories and flow field distributions under different sample flow rates at ***I_1*****.** In (**A**), (**B**), (**C**) and (**D**), the sample flow rate at I_1 was set to 50 μL/h. In (**E**), (**F**), (**G**) and (**H**), the sample flow rate at I_1 was set to 400 μL/h. For both simulations, the guide flow rates at I_2 and I_3 were set to 2000 μL/h and 3000 μL/h, respectively.

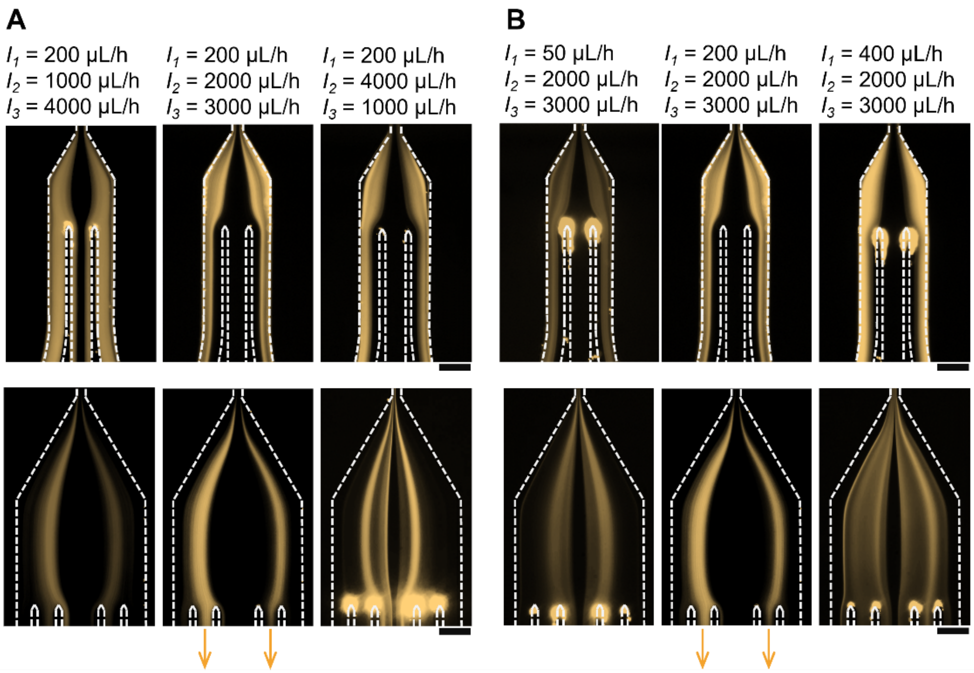

Fig. S8. Optimization of guide and sample flow rates for extracting 500 nm PS beads at specific outlets. Effect of guide flows I_2 and I_3 (total flow rate of I_2 and I_3 is set to 5000 µL/h) (a) and sample flow I_1 (b) on directing the 500 nm PS particles at desired outlets. When I_1, I_2, and I_3 are set to 200 µL/h, 2000 µL/h, and 3000 µL/h, respectively, almost all of the 500 nm PS beads exit through the desired outlets, as shown by the yellow arrows. Scale bars are 100 µm.

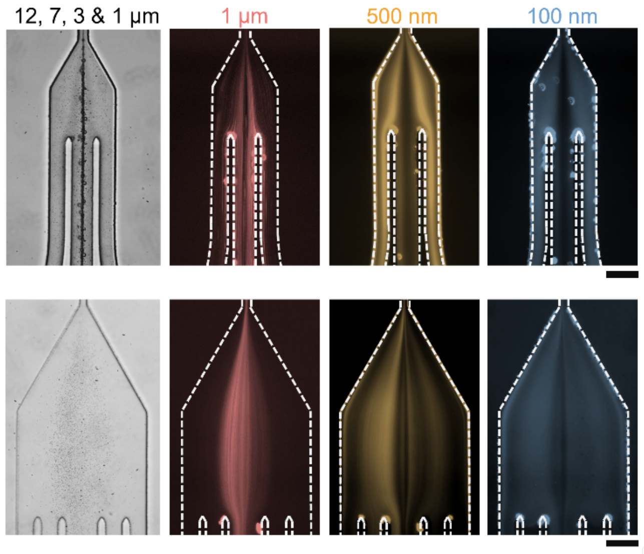

Fig. S9. Particle trajectories in the separation regions of both the cell-depletion (top row) and the sEV-isolation modules (bottom row) when particles are suspended in a 1 MDa PEO solution. The sample and guide flow rates for inlets I_1, I_2 and I_3, were set as 200 µL/h, 2000 µL/h, and 3000 µL/h, respectively. The corresponding 1 MDa PEO concentrations at inlets I_1, I_2 and I_3 were 0.1% w/v, 0.15% w/v and 0.1% w/v respectively. Particles with 500 nm and 100 nm diameters are distributed across the whole area of the sEV-isolation module indicating an inefficient particle separation. Scale bars are 100 µm.

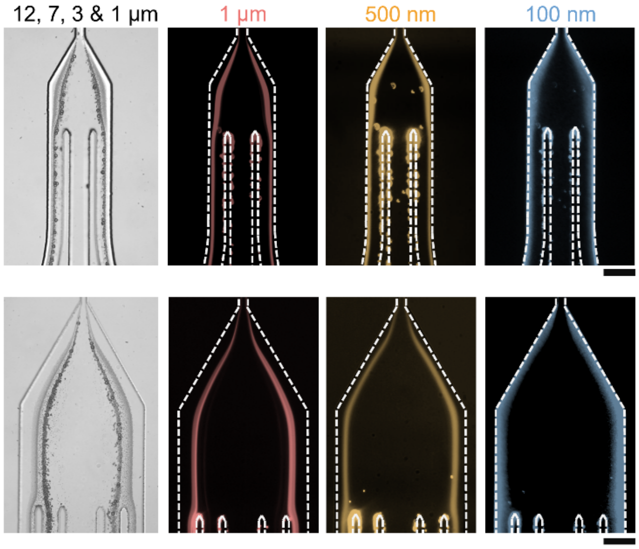

Fig. S10. Particle trajectories in the separation regions of both the cell-depletion (top row) and the sEV-isolation modules (bottom row) when particles are suspended in a 1× PBS solution. The sample and guide flow rates for inlets I_1, I_2 and I_3 were set as 200 µL/h, 2000 µL/h, and 3000 µL/h, respectively. In this case, inefficient separation of the 1 µm, 500 nm, and 100 nm particles occurs. Scale bars are 100 µm.

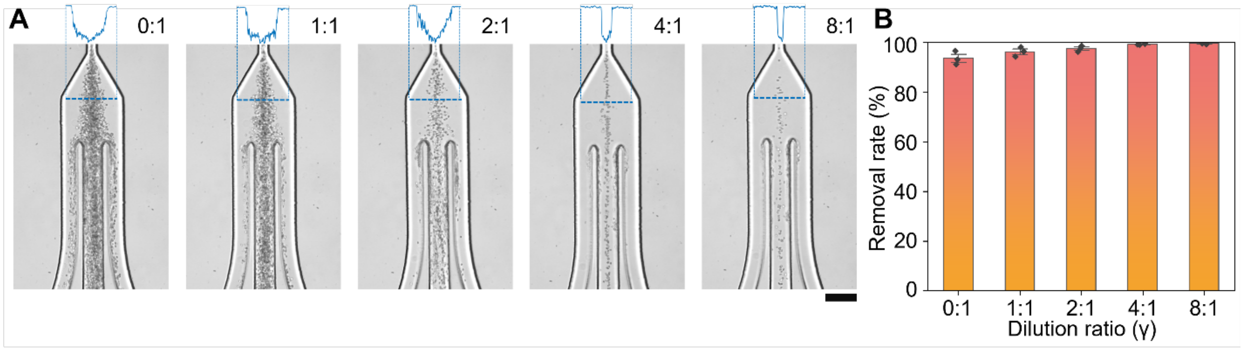

Fig. S11. Effect of the blood dilution ratio (γ) on separation performance. The sample and guide flow rates for inlets I_1, I_2 and I_3 were 200 µL/h, 2000 µL/h, and 3000 µL/h, respectively. (**A**) Blood cells were focused near the center of the channel at all tested blood dilution ratios. At low dilution ratios, the focusing width of the blood cell stream becomes wider, and thus more cells escape from the cell-depletion module into the downstream sEV-isolation module. (**B**) Removal rates of RBCs from outlet O_1 (n = 3 independent technical replicates, means ± SD). To balance separation efficiency with whole blood throughput, a blood dilution ratio 4:1 was chosen as the optimal dilution condition. The RBC removal rate is defined as the number of RBCs removed from outlet O_1 divided by the number of RBCs introduced through inlet I_1. Scale bars are 100 µm.

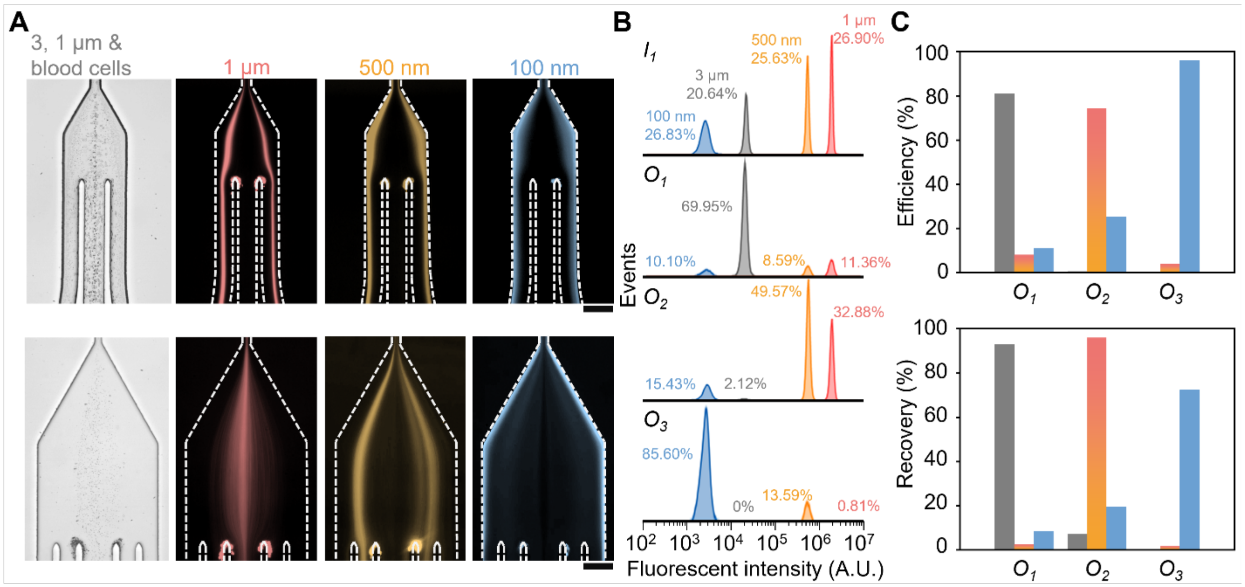

Fig. S12. Size-dependent separation of PS particles suspended in diluted blood (4:1 volumetric dilution ratio of 0.1% w/v 600 kDa PEO to whole blood). (**A**) Experimental observation of particle trajectories in the separation sections of both the cell-depletion (top row) and sEV-isolation (bottom row) modules. In the cell-depletion module (top row), blood cells and 3 µm particles are focused along the centerline and exit through the waste outlet O_1, whereas smaller particles (1 µm, 500 nm, and 100 nm) locate near the sidewalls before entering the sEV-isolation module. In the sEV-isolation module (bottom row), 1 µm and 500 nm particles are collected at outlet O_2, while the 100 nm particles are collected at outlet O_3. (**B**) Flow cytometry analysis of particle size distributions for the particle mixture at inlet I_1 and fluid collected at each outlet (O_1, O_2, and O_3). (**C**) Separation efficiencies and recovery rates calculated from flow cytometry data. In the case of 100 nm beads (representatives of sEVs), at the side outlet O_3, the separation efficiency and recovery are 96% and 72%, respectively. Scale bars are 100 µm.

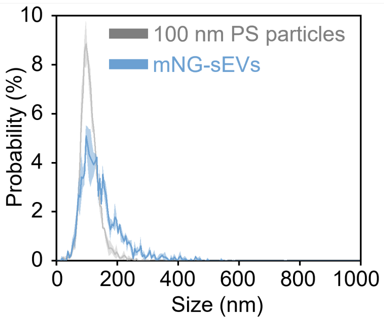

Fig. S13. NTA analysis of standard 100 nm PS beads, and mNG-sEVs. mNG-sEVs were extracted from cultured HEK293T cells using UC (n = 3 independent technical replicates, means ± SD).

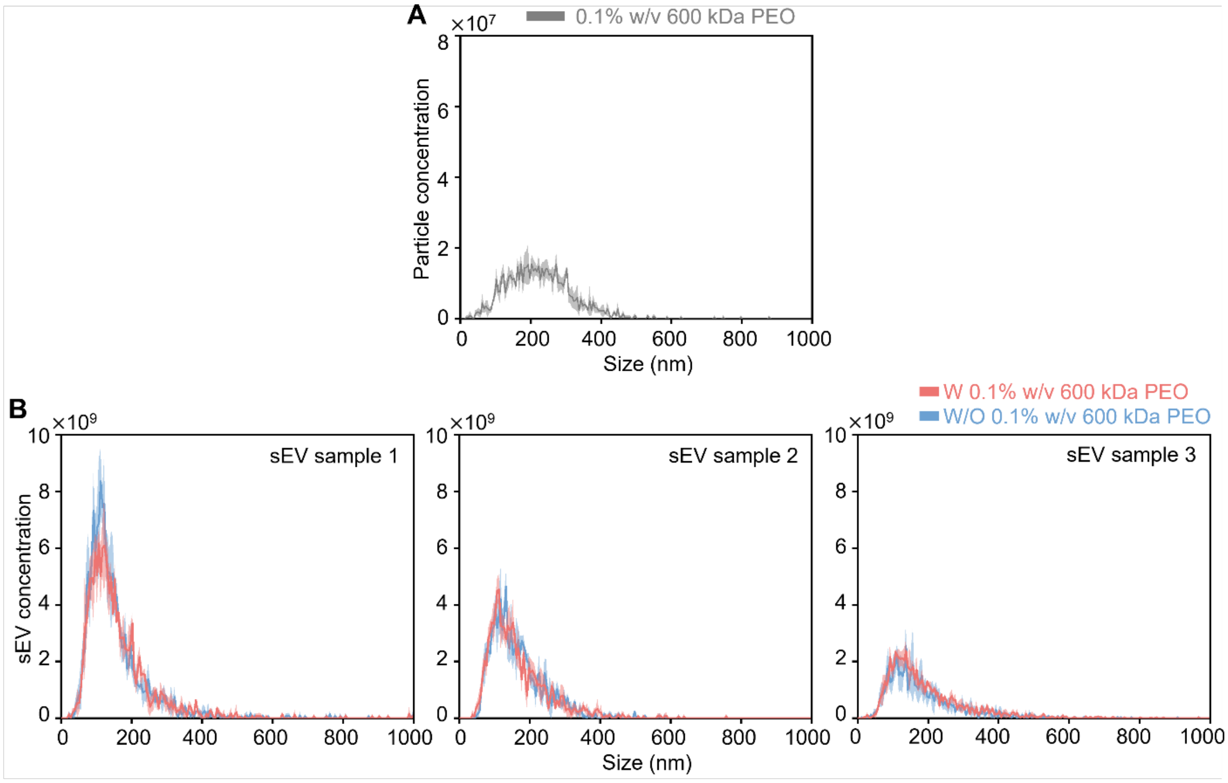

Fig. S14. Assessment of the impact of PEO on NTA measurements. (**A**) Particle size distribution and associated concentrations for a 0.1% w/v 600 kDa PEO sample (*n* = 3 independent technical replicates, means ± SD). (**B**) Particle size distributions and concentrations for three UC-derived sEV samples with and without 0.1% w/v 600 kDa PEO (*n* = 3 independent technical replicates, means ± SD).

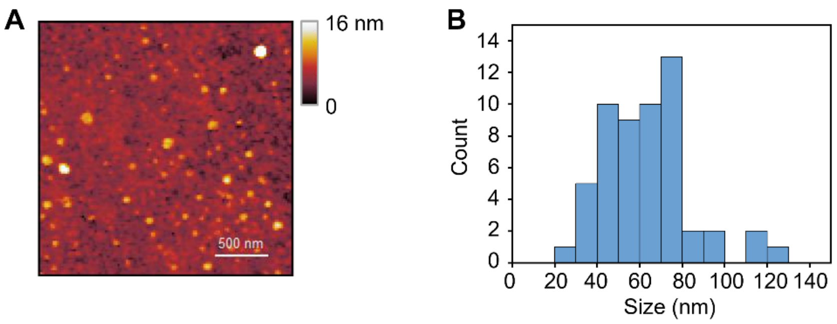

Fig. S15. AFM analysis of an sEV sample isolated from blood using the viscoelastic microfluidic device. (**A**) AFM image of the sEV sample. (**B**) Size distribution of the particles shown in the corresponding AFM image.

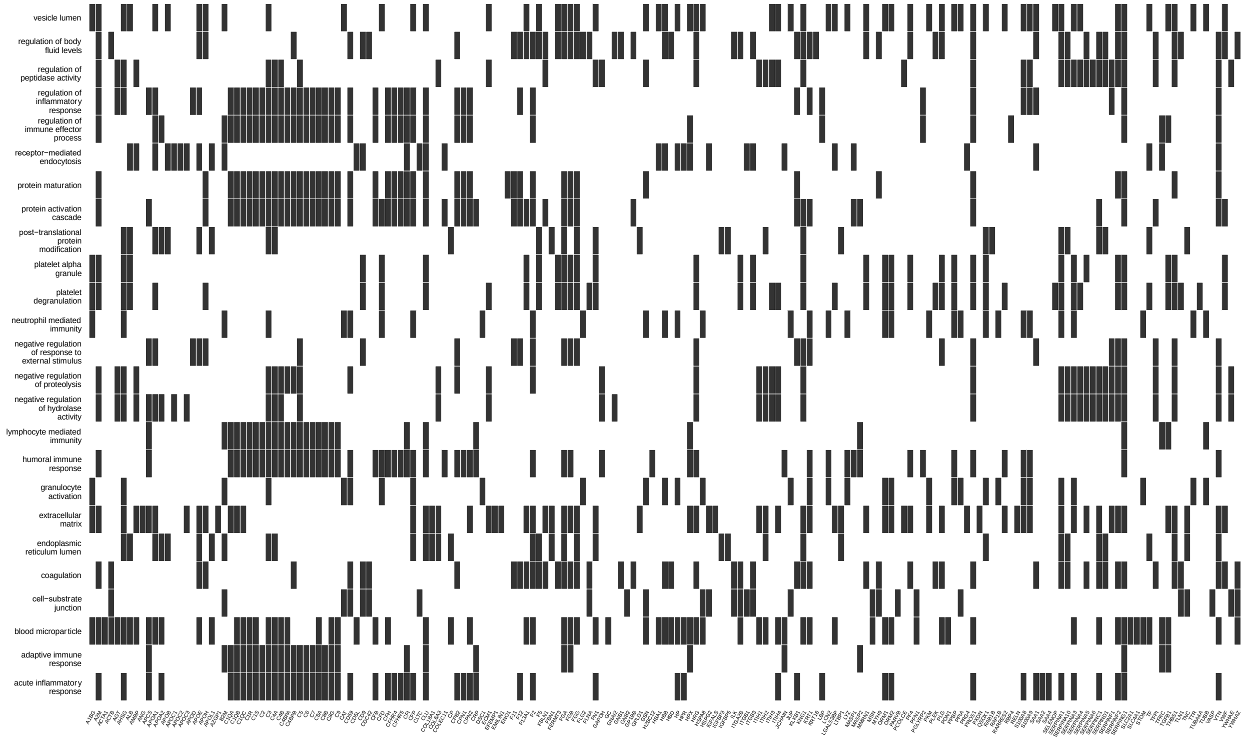

Fig. S16. **Pathway enrichment map of proteins detected in UC-derived EVs.** Black tiles indicate connections between individual proteins (x axis) and corresponding biological pathway annotations (y axis) according to pathway overrepresentation analysis (ORA).

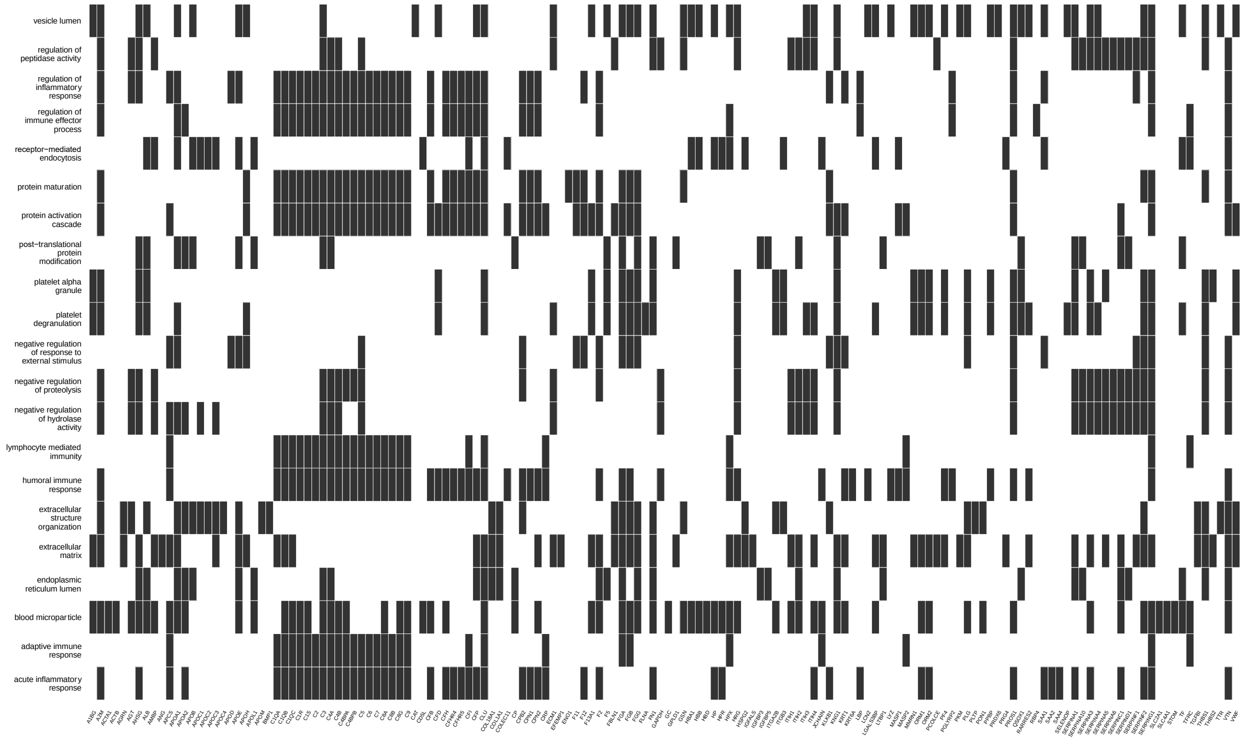

Fig. S17. **Pathway enrichment map of proteins detected in microfluidic-derived EVs.** Black tiles indicate connections between individual proteins (x axis) and corresponding biological pathway annotations (y axis) according to pathway ORA.

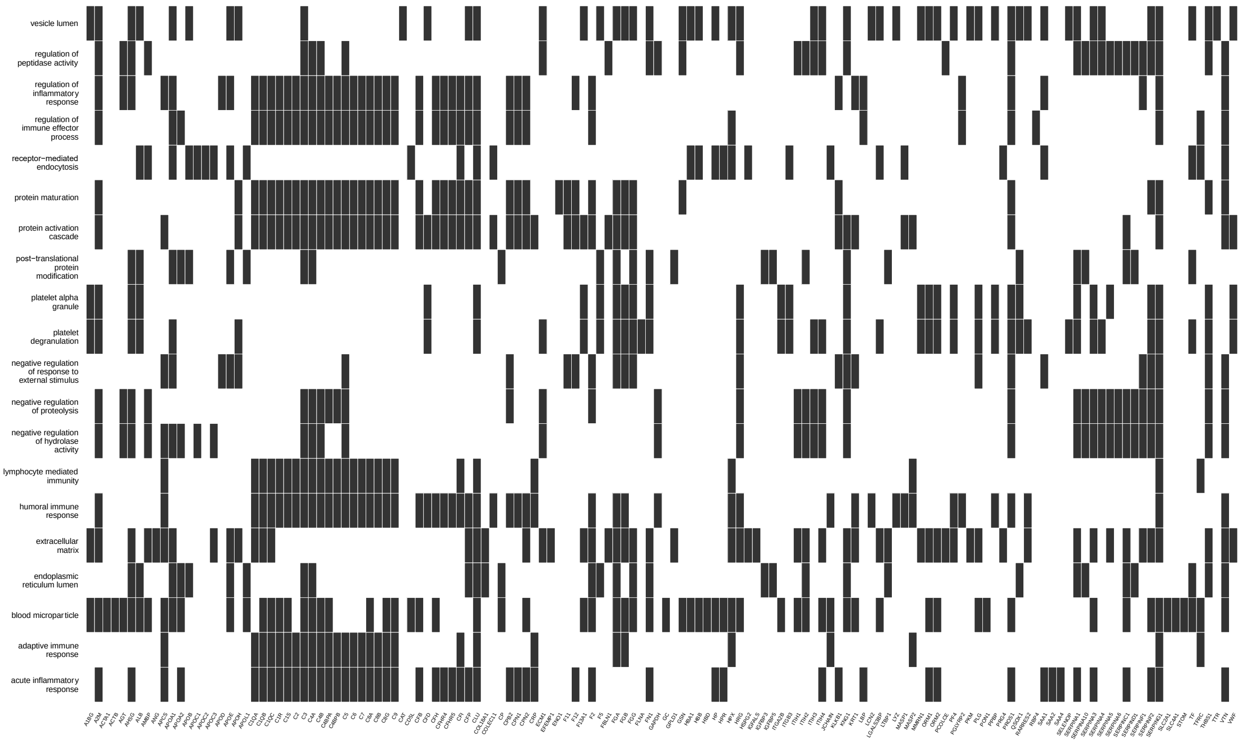

Fig. S18. Pathway enrichment map of overlapping pathways between UC- and microfluidic-derived EV proteomes. Significantly overrepresented pathways which are shared between both EV isolation methodologies are shown. Black tiles indicate connections between individual proteins (x axis) and corresponding biological pathway annotations (y axis) according to pathway ORA.

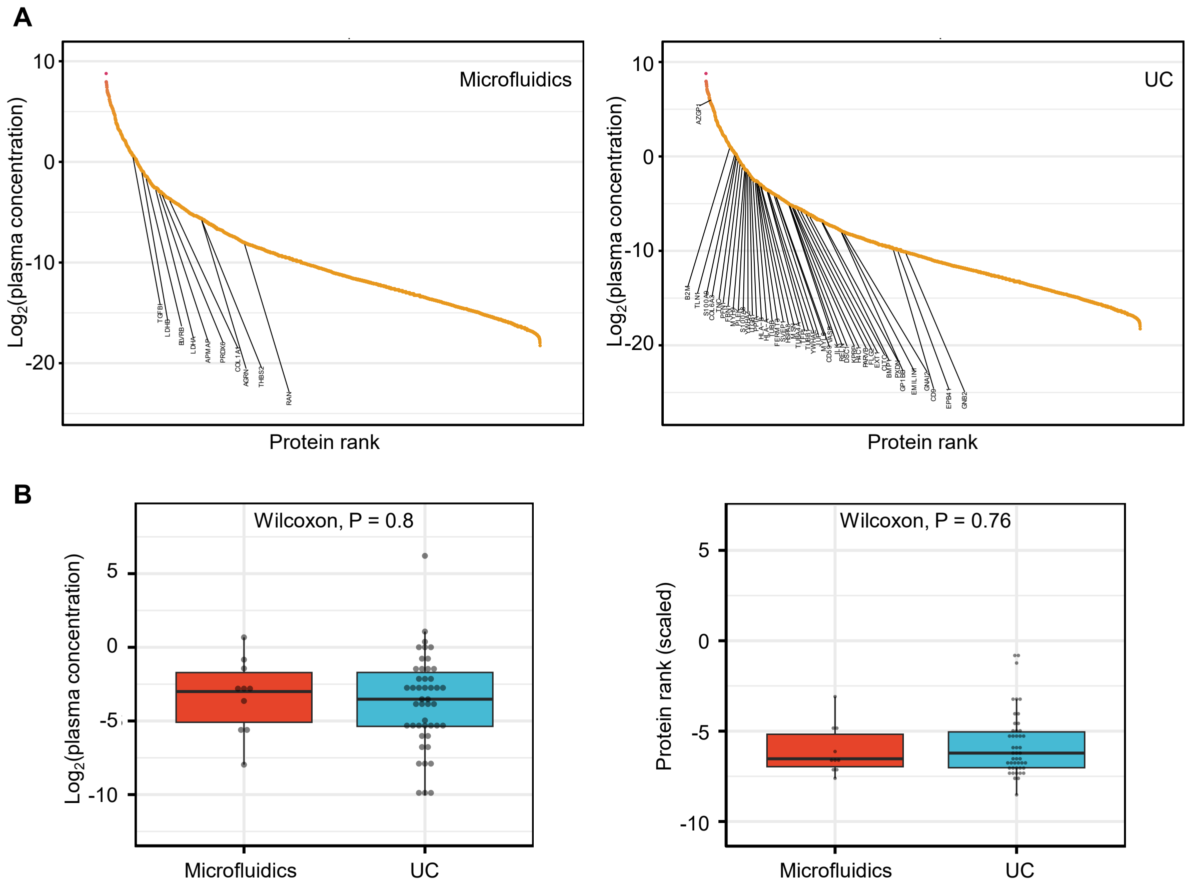

Fig. S19. Mapping of microfluidic- and UC-associated proteins onto the human blood plasma proteome dataset. (**A**) Mapping of the 10 microfluidic-associated (left) and the 48 UC-associated (right) proteins onto the quantitative human plasma proteome dataset derived from the HPA. (**B**) Statistical comparison of the abundance (left) and protein rank (right) distributions between microfluidic- and UC-associated proteins according to HPA dataset. The analysis indicates no obvious bias towards proteins of particular abundance regions. P values were calculated based on a two-tailed, unpaired Wilcoxon rank-sum test.

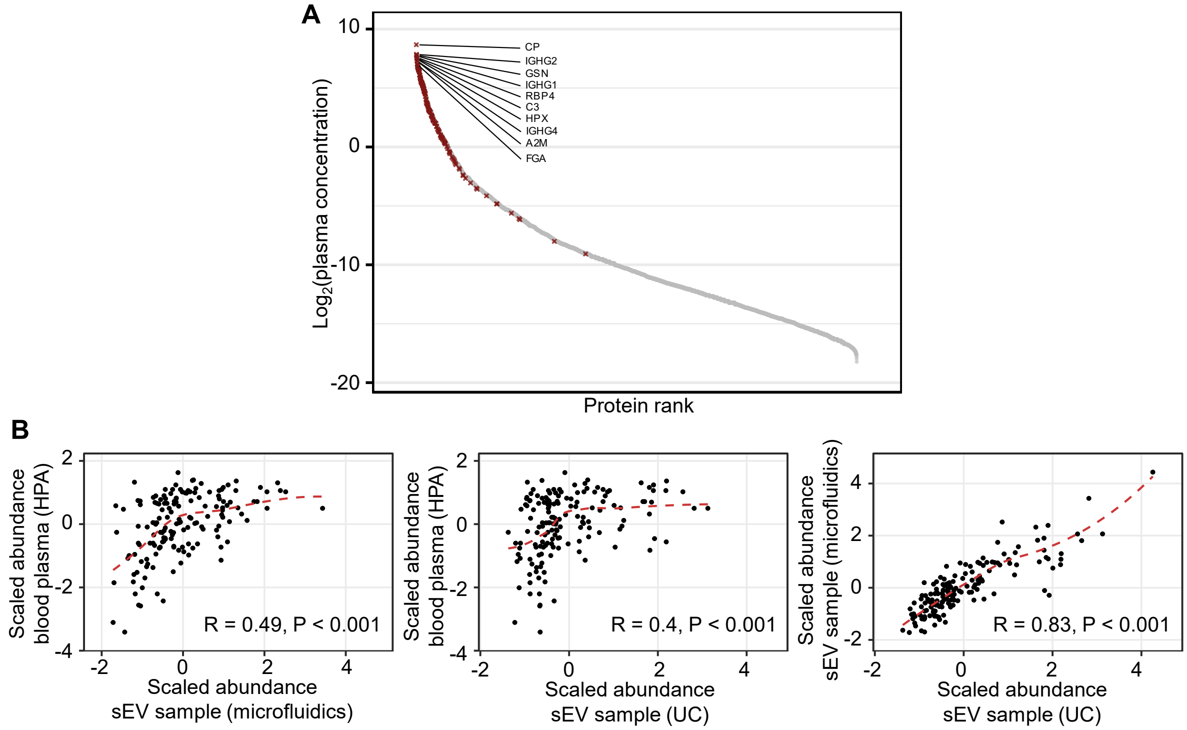

Fig. S20. Assessment of potential blood plasma contaminants in microfluidic- and UC-derived sEV samples. (**A**) Mapping of the 162 overlapping proteins (indicated by red crosses) shared between microfluidic- and UC-derived sEV samples onto the human plasma proteome dataset derived from the HPA. The top-10 microfluidic/UC-overlapping proteins are labelled. (**B**) Quantitative correlation of the microfluidic/UC-overlapping proteins with blood plasma data from the HPA dataset. Left: quantification based on microfluidic-isolated sEV samples; Middle: quantification based on UC-isolated sEV samples; Right: comparison between microfluidic- and UC-isolated sEV samples. Red dashed lines represent a local regression fit (LOESS). Regression coefficients (R) and P values were obtained from a Spearman regression model.

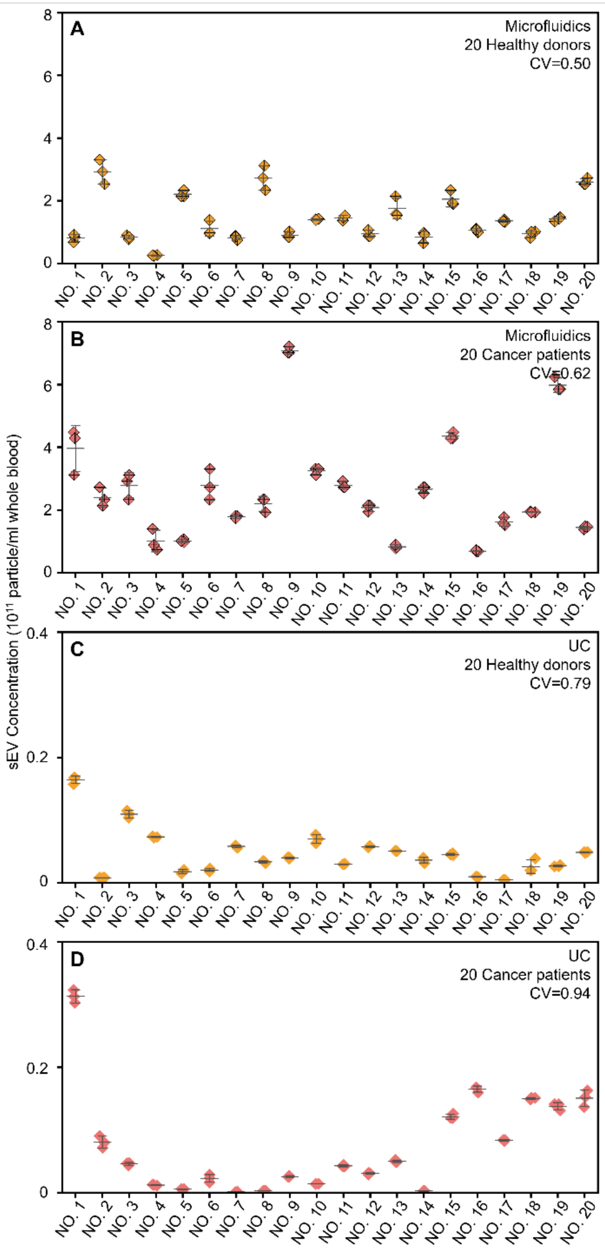

Fig. S21. NTA-based concentration measurements of sEVs isolated from Healthy Donors (HDs) and Cancer Patients (CPs). Concentration values (n = 3 independent technical replicates, means ± SD) of sEVs derived from both HDs and CPs using the microfluidic device (**A**) & (**B**) and the UC method (**C**) & (**D**). Samples with sEVs isolated using UC have a higher CV value, which can be attributed to inconsistencies in blood sample processing over the course of different days, causing batch-to-batch variations. To calculate the CV values, we first calculated mean values for each individual based on the three repetitions. Subsequently, CV values were determined based on the means of the 20 individuals within each group.

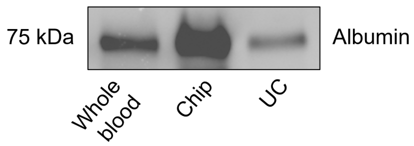

Fig. S22. WB analysis of the non-specific protein, albumin. A higher expression of albumin indicates a higher protein contamination.

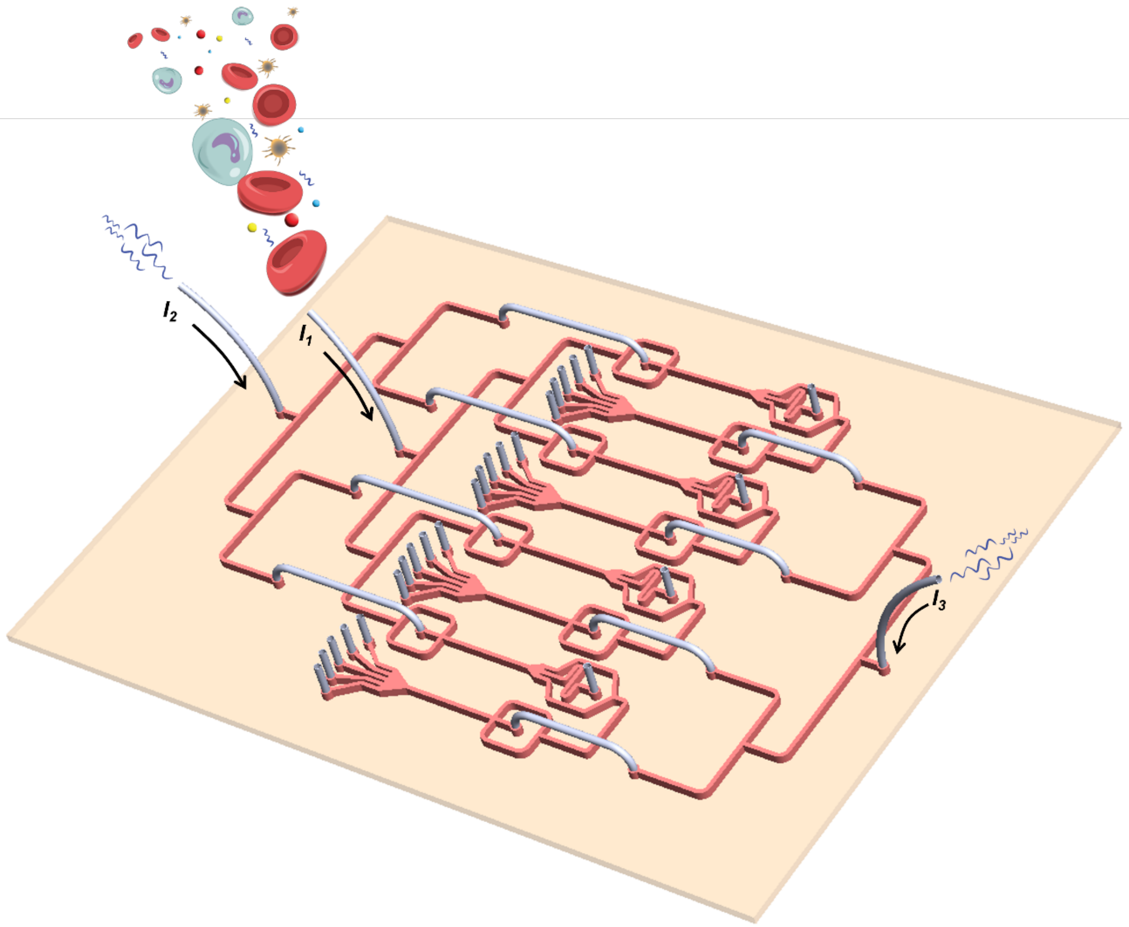

Fig. S23. Schematic of a multiplexing concept for scaling up the isolation of sEVs. Four of the current viscoelastic devices are integrated into one device for isolation of sEVs without increasing the complexity of the system (using 3 syringe pumps).

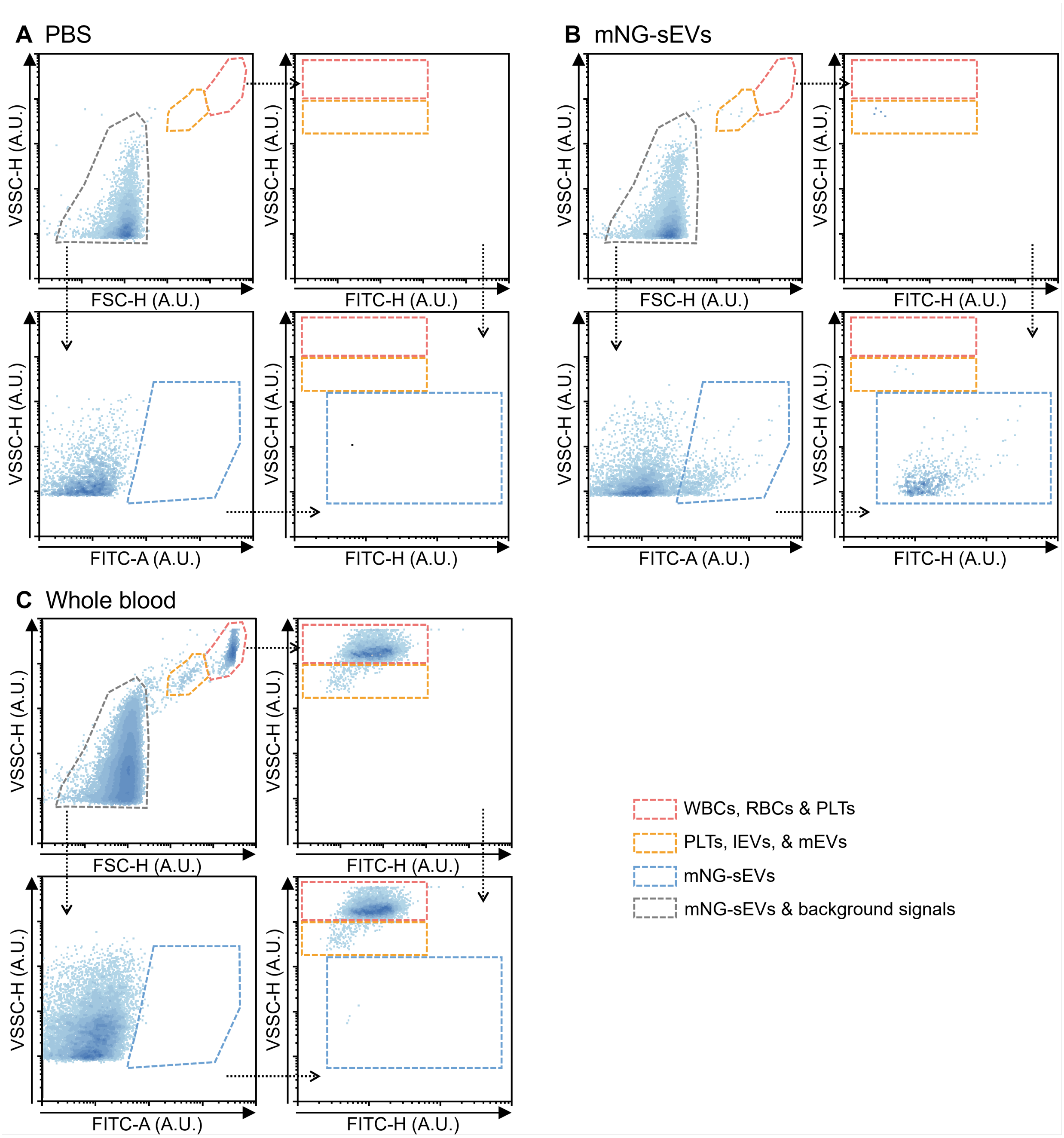

Fig. S24 Gating strategy used in flow cytometry analysis experiments. (**A**) PBS, (**B**) an mNG-sEV sample, and (**C**) a whole blood sample were measured separately by flow cytometry. WBCs, RBCs, PLTS, lEVs and mEVs were gated based on FSC-H and VSSC-H values. mNG-sEVs were gated based on FITC-A and VSSC-H values.

Table S1. Gender, age and cancer type information for 20 healthy donors and 20 cancer patients.

Healthy Donor			Cancer Patient			
No.	Gender	Age	No.	Gender	Age	Cancer Type
1	M	59	1	N.A.	50	Glioblastoma
2	M	62	2	N.A.	68	Glioblastoma
3	M	36	3	N.A.	42	Glioblastoma
4	M	32	4	N.A.	22	Glioblastoma
5	M	56	5	N.A.	86	Brain Metastatic Rectal Cancer
6	M	43	6	N.A.	66	Brain Metastatic Lung Cancer
7	M	33	7	N.A.	55	Glioblastoma
8	F	65	8	N.A.	83	Brain Metastatic Lung Cancer
9	M	24	9	N.A.	72	Glioblastoma
10	M	49	10	N.A.	63	Glioblastoma
11	M	22	11	N.A.	74	Glioblastoma
12	F	55	12	N.A.	34	Astrocytoma WHO 2
13	F	41	13	N.A.	63	Glioblastoma
14	F	62	14	N.A.	64	Brain Metastatic Lung Cancer
15	F	46	15	N.A.	82	Glioblastoma
16	M	20	16	N.A.	58	Brain Metastatic Rectal Cancer
17	F	21	17	N.A.	73	Glioblastoma
18	F	48	18	N.A.	58	Glioblastoma
19	M	52	19	N.A.	41	Astrozytoma WHO 3
20	M	57	20	N.A.	74	Endometrial Cancer

Table S2. Comparison of different microfluidic techniques for sEV separation from blood

Technique type	Throughput in whole blood (without parallelization)	Recovery	Purity	Flow	Advantage	Disadvantage	Ref.
Acoustic isolation	2.5 mm/s, ~0.014 mL/h	80%	N.A.	Continuous	Biocompatible; high recovery rate and purity	Complex device fabrication; requirements of external control	(46)
	0.24 mL/h	82.4%	98.4%	Continuous			(38)
Dielectrophoretic isolation	0.025 ml, 20min	N.A.	N.A.	Discontinuous	Controllable EV capture and release	Complex device fabrication; risk in electric/ thermal damage of sEVs	(45)
Immunoaffinity capture	3 mL/h	N.A.	N.A.	Discontinuous	High purities for specific sEV sub-populations	Expensive antibodies functionalization; harsh conditions for sEV release; low size resolution; low recovery rate; better for cell-free samples	(43)
Micro-filtration	0.24 mL, 2 h	N.A.	N.A.	Discontinuous	Field-free; easy operation	Complex device fabrication; risk in membrane clogging and sEV aggregation	(47)
	0.5 mL, 8 minutes	94%	N.A.	Discontinuous			(49)
	0.002 mL, 10 minutes	99%	N.A.	Discontinuous			(48)
	0.03~0.6 mL, 10~40 minutes	75%	N.A.	Discontinuous			(50)
	0.2 mL, 30 minutes	10.5%	N.A.	Discontinuous			(51)
Inertial isolation	1.2 mL/h	Low, ~16%	N.A.	Continuous	Field-free; low cost; simple device; easy operation.	Low size resolution; low recovery rate	(31)
Viscoelastic isolation	0.3 mL/h	N.A.	85.4%	Continuous	Field-free; low cost; simple device; easy operation.	Low size resolution; low recovery rate; polymer additive	(32)
Viscoelastic isolation	0.04 mL/h	87%	97%	Continuous	Field-free; low cost; simple device; easy operation; high size resolution; high recovery rate and purity	Polymer additive	This Study
